# A survey of security, privacy and trust issues in vehicular computation offloading and their solutions using blockchain.

**DOI:** 10.12688/openreseurope.16189.2

**Published:** 2023-10-25

**Authors:** Sharifah Yaqoub Fayi, Zhengguo Sheng

**Affiliations:** 1 School of Engineering and Informatics, University of Sussex, Brighton, England, BN1 9RH, UK; 2 School of Engineering and Informatics, University of Sussex, Brighton, England, BN1 9RH, UK

**Keywords:** Vehicular network, Security, Privacy, Trust, computation offloading, Blockchain, Edge computing, VEC

## Abstract

Continuous improvement in transportation systems and smart vehicles' appearance make new highly intensive applications. Complex applications need high-performance capabilities, real-time responses, and generate massive amounts of data to process and exchange. This presents the idea of vehicular edge computing (VEC), which is proposed to handle complex applications and satisfy smart vehicle processing requirements. VEC enables computation offloading to an edge server to reduce communication latency, execution cost and energy consumption greatly. However, offloading to another node opens up new vulnerabilities regarding security and privacy. Moreover, trust issues in such an untrustworthy environment need an effective trust management solution and incentive mechanisms to improve overall security. This will increase the computation offloading success rate and the vehicles' willingness to share their resources. Particularly given the high transportability and heterogeneity of vehicular networks, the conventional security and trust management methods are inadequate. Blockchain, the rapidly emerging trend technology, is a unique solution that can help overcome security and privacy issues and meet trust management and incentive mechanism goals. Blockchain’s immutable distributed ledger, traceability, consensus validation system and smart contract features can improve vehicular network security. Although most research is focused on enhancing the performance of computation offloading algorithms, blockchain security solutions in computation offloading scenarios are not fully discussed. Thus, security and trust issues related to computation offloading in VEC environments need more consideration since supporting the new complex vehicular applications is essential. Therefore, this paper provides a review of recent surveys and studies, an overview of VEC, computation offloading and blockchain, in addition to discussing security, privacy and trust in vehicular networks and computation offloading while considering blockchain as a distributed security solution. We propose a new paradigm called blockchain edge of vehicle (BEoV) at the end, which enables several blockchain-based security services for vehicular computation offloading in particular.

## Plain language summary

As transportation systems and smart vehicles become more advanced, new intensive applications are made possible. Vehicular Edge Computing (VEC) has been proposed to allow vehicles with overloaded resources to delegate computations to a server located at the edge of the network, edge servers. This reduces communication latency, execution costs, and energy consumption, but introduces new security and privacy risks. Blockchain is a possible solution to improve overall security, increase the success rate of computation offloading, and increase the willingness to share free resources. Recent studies focus on improving computation offloading performance methods, but blockchain security solutions aren't discussed enough. This paper reviews recent surveys and studies, gives a background overview of computation offloading and blockchain, and discusses security, privacy and trust in vehicular networks and computation offloading while considering blockchain as a possible security solution. We're introducing a new paradigm called Blockchain Edge of Vehicle (BEoV) at the end, which allows several blockchain-based security services for vehicular computation offloading.

## Introduction

Through decades, transportation systems have been developed. However, the number of vehicles is increasing, and it is expected to be around two billion by 2035 ^1^. This enormous growth increases road congestion, pollution, accidents and their caused deaths ^2^. Because of these unpleasant effects, new changes in transportation systems should be considered ^3^.

The Internet of Things (IoT) has been considered the beginning of the upcoming of the internet era revolution allowed by machine-to-machine (M2M) communications. We can utilize the emergence and popularity of IoT and the future of ubiquitous computing in building practical intelligent transportation systems (ITS) ^4^. The conversion to ITS will save our society by developing road safety and driving methods in addition to providing infotainment to drivers and passengers ^5^. To achieve the transportation conversion goal, vehicular *ad-hoc* networks (VANETs) appeared to build a vehicular network that supports the network’s nodes' communication and driving-related information sharing ^5^. In VANETs, vehicles share and exchange information about their speed, location, and road conditions to ensure awareness of their surroundings and make efficient decisions based on that knowledge ^6^.

However, the increase in these vehicle quantities and their generated data require more intensive computation and high-performance resources than what is already equipped. As a result, there is a need to improve the vehicular network environment and to develop vehicle capabilities in order to move toward intelligent and autonomous vehicles ^7^. The Internet of Vehicles (IoV) is the upcoming efficient branch of the IoT in the transportation department that enables vehicles to move to ITS ^2, 8^. Nevertheless, it is more complex in its idea of containing an array of sensors and stakeholders, and hence it has some different features that distinguish it from other IoTs ^9^.

IoV is a distributed network that provides communication among different parts of the network, for example, vehicles, roadside units (RSUs), sensors, and humans ^3^. Besides all the vehicular network aspects currently in VANETs, IoV targets the intelligence of vehicles. Therefore, VANETs are considered one of the IoV components ^7, 10^. In short, IoV operates the future of two evolving technologies, vehicle networking and vehicle intelligence ^3, 7^. 

This intelligent network provides high computing and communication abilities that facilitate more efficient and safer applications for vehicles and humans related to them ^7^. Exchanging data and resources among smart vehicles in a seamless and connected environment is the primary aim of IoV ^8^. The continuous improvement in the IoV field and the appearance of smart vehicles make new highly intensive tasks and applications like autonomous or self-driving ^8, 10^. Such complex applications need high-performance capabilities and real-time responses besides generating massive data to process and exchange. However, the computational limitations of the onboard resource vehicles make it extremely difficult for the vehicles to manage such complex tasks and to meet their requirements in terms of resources and quality of experience (QoE) ^11^.

Cloud computing has been used in the past and to this present day for a considerable period as the infrastructure management of intensive computation applications and massive data. However, it demands a long geographical distance that makes response delay and bandwidth expensive, which are not acceptable in real-time applications and time-sensitive tasks ^11^. Therefore, cloud computing is not suitable to support such types of applications.

The edge computing concept has appeared to overcome cloud computing issues. The computation resources are placed close to the clients at the network’s edge to support dynamic scalability and improve network performance. Regarding this, multi-access edge computing (MEC) concept has emerged to extend the cloud infrastructure. Through the use of this concept, performance is improved, latency is reduced, and energy is saved by offloading computation to edge servers ^11^.

Considering all the discussion mentioned, much research is being conducted on the integration of the MEC concept into vehicular networks, therefore such integration presents the idea of vehicular edge computing (VEC) ^12^. VEC has appeared to meet the computation demand of smart vehicles and to deal with complex applications, which are “computation-intensive and time-constrained tasks”, in vehicular network applications ^11, 13^. Furthermore, VEC supports different application scenarios, including road safety, traffic control, autonomous driving, video streaming, and data aggregation and mining ^12^. VEC comprises a computation server often integrated with RSU close to vehicles. This architecture facilitates communication and computation among the networks. Consequently, it helps in mitigating the latency by avoiding contact with the distance central cloud. Mitigating the delay time in vehicular networks is a demand in such a dynamic environment ^13^.

One of the practical applications of VEC is enabling computation offloading. Offloading the computation-intensive tasks to the edge servers or other nodes reduces the communication latency, execution cost and energy consumption vitally ^11, 12, 14, 15^. Also, it helps to mitigate network congestion and the node’s load work ^11^. For example, a vehicle that suffers from a resource shortage can use offloading to transfer a task to a nearby VEC server, either RSUs or base stations (BSs), to process it and return the result to the vehicle ^13^.

Since offloading computation consumes more energy and time than performing the tasks locally, it is vital to decide when it is required to offload. Deciding the optimal solution regarding the extra computation cost and the improved performance is mandatory ^11^. Therefore, latency and computational power must be well-adjusted ^16^. Additionally, offloading to another node opens new vulnerabilities regarding security and privacy. Moreover, to promote resource sharing and attract vehicles to engage in the offloading process, trust management and incentive mechanisms in such a low-trust environment need to implement ^17– 20^.

Security, privacy and trust are three critical issues in all vehicular network applications that need to be considered ^5^. These significant challenges can affect the rapid evolution of all IoV applications including VEC. Investigating these three aspects is a continuing concern within vehicular computation offloading. Therefore, more studies are required to provide new secure communication methods in vehicular networks, including computation offloading ^8, 16^. The traditional trust and authentication methods are incompetent, especially with high mobility and heterogeneity. The research concerning this challenge in VEC is at the beginning stages and needs more significant efforts to deal with this issue efficiently ^12^.

Blockchain, the new emerging trend technology, is a unique solution that can help overcome security and privacy issues and meet the trust management and incentive mechanism goals ^13, 17, 21^. Blockchain and VEC have the same concept of distribution framework. However, the vehicular network can inherit unique blockchain features that help overcome security, privacy, and trust issues, specifically in computation offloading cases. Decentralization, transparency, immutability and traceability, consensus mechanisms and smart contracts functionality are beneficial blockchain features for securing VEC ^13, 17, 21^. Furthermore, different security aspects like access authentication, data privacy, attack detection, and trust management in applications such as smart transportation and vehicular networks can be achieved by utilizing blockchain ^21^. 

Despite all of the great advantages of blockchain, integrating it with VEC proposes certain challenges due to the unique features of the vehicular network. In addition, motivating vehicles to contribute to the computation offloading requests and ensuring the reliability of resource allocation are issues that need to be shed light on. Therefore, blockchain integration with IoV applications, such as in the VEC environment, has been focused on recently ^13, 17^. However, in the context of computation offloading, most researchers assume the vehicles’ trustworthiness and do not consider security and privacy issues ^17^. Therefore, this emerging integration significantly requires more research and studies to overcome the difficulties in security, privacy, and trust aspects of vehicular networks and particularly in the case of computation offloading, which will be the primary concerns of this paper.

### Contribution

We assess current related research that has been stated in various works of literature with a specific emphasis on the security of VEC in computation offloading situations, we review state-of-the-art studies on the deployment of blockchain for vehicular network applications and show the lack of the main consideration of security in vehicular computation offloading scenarios. In this survey, we generally focus on vehicular networks security issues and requirements besides the role of blockchain in supporting security in various vehicular networks and computation offloading applications, in particular. The contributions of this survey are summarized as follows.

1. We provide a comprehensive review of the preliminary background, which includes VEC, computation offloading and blockchain technology aspects.

2. We discuss the challenges faced the vehicular networks in terms of security, privacy, and trust in general and in computation offloading cases, we emphasize both the importance and motivations of blockchain and VEC integration and highlight the advantages of employing blockchain as a distributed security solution

3. We analyze some possible integration challenges and outline several open issues as future research opportunities in blockchain-based computation offloading scenarios and based on the survey we introduce a new paradigm called Blockchain Edge of Vehicle (BEoV) that can provide several blockchain-based security services for securing vehicular computation offloading specifically and point out a suggested solution for trust management in the computation offloading scenario.

**Figure 1.**
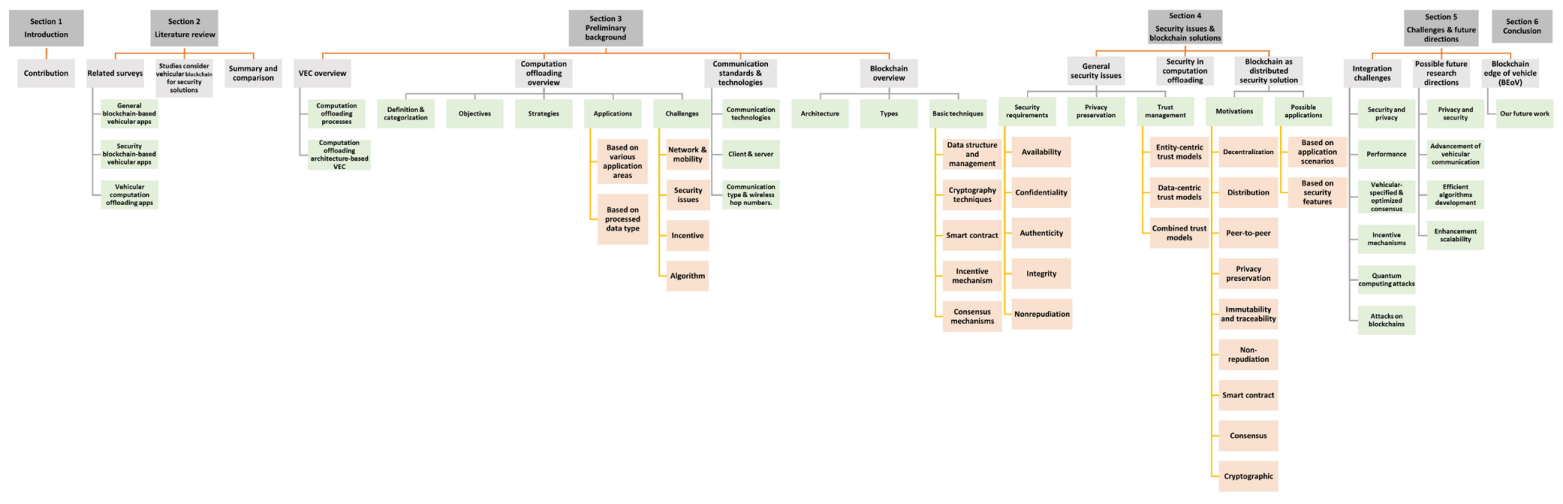
Taxonomic view of the survey.

The remainder of this paper is organized as follows: Literature review section provides assessment of related survey papers and various studies then the Preliminary background section discusses the background and preliminary aspects related to the VEC, computation offloading and the blockchain overview. Third section analyses the security in vehicular networks by first discussing security, privacy, and trust issues in vehicular networks besides considering computation offloading in edge environment-related security issues. In addition, it discusses blockchain technology as a promising security solution by showing the motivation to integrate blockchain and exploring the different security blockchain-based applications categorized into two categories. Finally, the integration of the blockchain challenge is discussed in the third section and mentions some possible future research directions besides explains the new paradigm Blockchain Edge of Vehicle (BEoV) with our future work and the suggested solution. Figure 1 illustrates the taxonomic view of the organization of this survey.

## Literature review

Throughout the literature review, initially, the recent researchers have focused on the recent advances in vehicular networks ^5^, more importantly on security issues ^22^. Additionally, they have concentrated on blockchain technology surveys that give an overview and explain new opportunities rather than cryptocurrencies and finance to leverage it like IoT applications ^23– 25^. Then the core of the research moves toward blockchain-based vehicular applications and mainly on security services offered by blockchain to overcome security, privacy, and trust issues in vehicular networks. Following a review and discussion of the recent related surveys and then a specific review of current studies related to vehicular blockchain-based security solutions.

We categorize 25 surveys into three groups based on their main objective as following:

***General blockchain-based vehicular applications.*** Some surveys have recently addressed blockchain-based vehicular applications generally, such as ^8^, which can be considered one of the leading surveys correlated to understanding the integration of blockchain and IoV overall aspects. Moreover, the survey mentions the security issues in computation offloading as a future direction that needs further investigation. Conversely, the research focuses on greener communities, smart grids, electric vehicles (EVs) and charging cases in ^26^. Both surveys briefly examine the challenges and limitations of the combination of blockchain with IoV like privacy, security, scalability, and incentive mechanisms. Recent surveys such as ^27^, examine the potential blockchain applications for ITS in general and blockchain-based IoV (BIoV) scenarios particularly using recent analysis and explanatory tables. Whereas the most recent one ^28^ targets the related aspects of blockchain applications in cooperative intelligent transport systems (C-ITS) in detail and provides four categorizations for the recent surveys and qualitative analysis of the mentioned proposals.

On the other hand, a survey like ^29^ presents a thorough taxonomy of adopting blockchain and edge computing in IoV and provides a comparison among various proposals based on access technology, IoV architecture layers, networking technologies, blockchain layer execution, and machine learning (ML) algorithms applicability. Also ^30^, observes the security aspects of edge-centric IoV regarding security attacks and requirements in each communication type, reviews some remediations and recommended future directions, including utilizing scalable blockchain-based IoV systems, artificial intelligence (AI) and ML algorithms. On the other hand, surveys like ^12^, concentrate on the VEC paradigm which broadly surveys VEC in different aspects, reviews enabler technology, analyzes various application scenarios and recent models such as task offloading and discusses the security and privacy challenges besides blockchain as a protection scheme enabler. Similarly ^31^, provides an overview of the VEC but more comprehensively, focusing on security in more detail regarding threats, authentications and privacy.

***Security blockchain-based vehicular applications for objectives.*** For example ^32^, surveys the recent blockchain-based cybersecurity applications for vehicular networks and analyzes them based on communication, authentication, trust management and privacy. They recommend more studies abruptly to improve vehicular network security since it is still at the early stage. However, they don’t include emerging technologies like 5G and machine learning. Differently in ^33^, various blockchain-based IoV applications are categorized and reviewed to improve security, privacy, trust and cooperation besides recommending the integration of new technologies like AI and edge computing in blockchain-based vehicular networks. Moreover, a survey such as ^34^ examines various security threats in IoV and discusses recent studies of blockchain techniques to secure IoV based on seven categorizations, including security, trust, authentication, data-sharing, decentralization, distribution, reputation, and privacy-based techniques. Additionally, seven possible methods that IoV and blockchain technology can be combined are discussed in ^35^, including preserving privacy in IoV, certificate management, trust management, blockchain-based IoV security and blockchain-based revised IoV architecture, besides considering the integration of new technologies like software-defined networks (SDN) and AI.

In contrast, the most recent survey ^36^ analyzes the security aspects of using blockchain with VANETs. It gives an overview of VANETs’ security challenges and attack classifications and discusses blockchain-based approaches for VANETs by considering seven security aspects like attacks, authentication, access control and key management. Moreover ^37^, is one of the early comprehensive surveys that discusses the security, privacy and trust management blockchain-based solutions in various vehicular networks like VANETs, IoV and smart vehicles with considering new supportive technologies such as 5G and edge computing. It discusses the appropriate blockchain types and consensus in the vehicular environment and mentioned some challenges and open issues. However ^6^, particularly surveys a broad selection of current blockchain-based authentication solutions in IoV and VANETs besides discussing the security attacks and requirements in IoV, while ^18^ focuses the survey on the security and privacy features regarding the vehicle to everything (V2X) communication in IoV and discusses the potential integration of blockchain with V2X technology and some vulnerabilities like 51% attack and smart contract exploitation. Additionally, the current comprehensive survey ^38^ provides a broad range of analyses for various last blockchain-based security schemes in vehicular networks based on two perspectives, applications such as transportation and resource sharing and security such as security requirements, network attacks and authentication techniques. Moreover, it examines the role of emerging technologies like edge computing, unmanned aerial vehicles (UAVs) and 5G in addition to discussing the significant challenges and future directions.

***Vehicular computation offloading applications.*** Other surveys aim to concentrate on one application enabled by edge computing which is computation offloading such as ^39^ surveys data offloading techniques in VANETs and explains the importance of offloading through VANETs by different communication modes, V2V, V2I and V2X and presents security issues and cluster challenges as possible future work. In contrast ^40^, specifically surveys the computation offloading concept, problems, and schemes for the VEC environment but concisely, while ^16^ examines the computation offloading for vehicular networks in a comprehensive and organized way to guide researchers in vehicular computation offloading environment. It differentiates the several concepts related to cloud, edge, and fog computing in vehicular networks with the help of a figure that clearly showed these differences. Moreover, the classification of research in vehicular computation offloading is based on three broad aspects, communication standard, problem, and experiment, which are classified into more specific topics. Nevertheless, in the survey, there is no specific object that analyses the security enhancements, and they highlight security and privacy as an open challenge and future direction opportunity. Also, it just mentions using blockchain once.

Relatedly ^20^, surveys the computation offloading in VEC in a taxonomic view with considering content caching and delivery too. It provides an overview of the VEC and its architecture of four layers and illustrates this clearly. It uses the paradigm (ComOf) for computation offloading and (CachDel) for content caching and delivery and discusses those computation types in different aspects systematically. It examines offloading and caching security issues and mentions blockchain as a security scheme. Also, in the open issues and future works, it mentions security and privacy. In contrast ^41^, observes the computation offloading for VEC by considering the output data retrieval algorithms, models, and classification. The taxonomy classified the recommended solutions for computation offloading by considering the crucial computation offloading steps, partitioning, scheduling, and data retrieval. They suggest considering ML for task partitioning and assignment and integrating VEC and 5G as future directions. Likewise ^42^, observes the computation offloading algorithms in the VEC networks but focuses on various computation offloading schemes, including learning-based, game-based, mobility-based, joint offloading, delay optimization and energy efficiency. On the other hand ^43^, gives a thorough analysis of different computing paradigms that allow task execution and computation offloading in VEC like MEC, VCC, and VEC. Differently ^44^, provides a brief literature review comparing blockchain technology’s significant contributions to the vehicular environment.

## Studies consider integrating blockchain as security solutions

Based on all the unique features that enable blockchain to be a promising solution to overcome the security issues, various studies have considered integrating blockchain into vehicular networks to handle security, privacy, and trust issues. For example, studies propose models based on blockchain to secure various vehicular network scenarios, such as ^45^, which utilizes blockchain to enable security and privacy for IoV in message dissemination scenarios and ^46^ considers optimizing the consensus management by designing reputation-based voting mechanism and contract theory while ^47^ proposes an improvement authentication mechanism for IoV based on blockchain. Moreover, some studies focus on integrated blockchain in VEC scenarios like ^48^, which proposes a trusted distribution mechanism of tasks to ensure fairness of distributing tasks and data integrity. The proposed system combines the scoring model, reputation model and incentive mechanisms besides proposing a multi-weight consensus algorithm. 49 which proposed a blockchain model focused on information and energy security interactions in electric vehicles cloud and edge computing and defined data and energy coins. 50, 51 and ^52^ focus on proposing a secure and effective solution for data sharing in VEC by utilizing a consortium blockchain. 50 utilizes (DSSC) and (ISSC), which are two smart contracts, one for data storage and the other for information sharing that curry the consensus mechanisms and develops a reputation-based data-sharing scheme with three-weight subjective logic (TWSL) model. 51 considers the combination of 5G and blockchain that makes sharing data based on the score model without using RSUs possible and designed an improved DPoS consensus algorithm. At the same time ^52^, utilizes smart contracts to guarantee a trustworthy environment for securing data storage and share, applies PBFT consensus and enhances reputation-based data management’s security. Moreover ^53^, mainly aims to secure blockchain-based emergency-driven message protocol for 5G enabled VEC. They utilize private blockchain and provide security analysis and an adversary model.

On the other hand, many studies specifically propose security solutions for computation offloading in edge environments. For example ^54^, provides a reputation blockchain-based model where RSUs offload tasks to adjacent fog vehicles based on repute scores and current workload. Also, it utilizes proof of elapsed time (PoET) consensus to select a leader, and a voting mechanism to validate the block of the consortium blockchain. 55 uses consortium blockchain, BFT-based PoS consensus and smart contract for secure resource sharing in VEC against the malicious behaviors of participants. Furthermore, they present a contract-based incentive scheme to motivate vehicles to share their computation resources, provide security analysis and consider using DRL in future work for resource-sharing schedules. 56 proposes a secure and scalable framework for blockchain-based edge computation offloading in social internet of vehicles (SIoV). They present an excellent security analysis with more intricate details and propose a dynamic PoW consensus (dPoW) with a checkpoint mechanism and a resource assignment policy. However, several research works were more specific and concentrated on one security aspect, which is authentication, since authentication plays a vital role in a vehicular network due to the underlying dynamic topology with vehicles joining and frequently leaving, for instance ^47, 57– 60^ provide frameworks for authentication in the vehicular network. Regardless, it is worth mentioning the most recent study ^61^ that aims to enhance the performance QoS of distributed edge computing with blockchain technology to enable ultra-reliable low-latency V2X communications and energy-efficient offloading by proposing multi-chain architecture based on the main chain, which manages multiple semi-independent segments.

## A summary and comparison of the state-of-the-art

To sum up, almost all mentioned surveys agreed that designing an effective, lightweight blockchain system in such a dynamic environment is challenging regarding scalability, suitable consensus, and incentive mechanism. Moreover, blockchain has been widely used as a security and trust solution due to the security services offered by blockchain that can support various aspects like authentication and access control. However, this integration faces new vulnerabilities in security and privacy, such as 51% attack and quantum attack, besides other issues like scalability ^38^. Through research and observation, it has been found that few surveys discuss security issues as a primary object and most other researchers mention security and privacy as an issue that needs to be considered, whether in vehicular and edge networks generally or vehicular computation offloading particularly ^5, 7, 8, 10, 12, 22, 35^. Moreover, various solutions are recommended regarding authentication and authorization, access control, and encryption. However, integrating blockchain technology is one of these solutions that got the attention of major researchers recently ^8, 17, 18, 20, 21, 25, 37, 38, 45, 62, 63^. Therefore, most surveys recommend that security, privacy, and trust require more studies when considering the integration of edge computing and blockchain that has not been investigated thoroughly for VEC computation offloading scenarios specifically.

Despite all the mentioned recent research efforts and with the high mobility of vehicles and continuous advancement of VEC, there are still significant research challenges and open issues facing the computation offloading in a blockchain-based vehicular network. Notably, security and privacy issues, besides trust problems, are critical concerns that will impact vehicular networks’ wide deployment and fast development. For instance, implementing encryption or authentication may negatively affect offloading effectiveness, quality of experience (QoE), and system scalability because processing tasks will take more time or demand more computational resources. However, these restrictions may be alleviated by blockchain technology with potential scaling solutions like sharding or sidechain structures. Additionally, there is a probability that prospective servers could refuse service to unknown requesters, which could result in failed offloading or underutilization of network resources. Therefore, applying an efficient Incentive mechanism is demanded to overcome this issue. Furthermore, reputation score management and data reliability can also help tackle malicious nodes that try to upload tasks containing viruses, ensure secure communication, and avoid spreading viruses or false information. Finally, a verifiable computing method for vehicular users, such as zero-knowledge proof, is required to prove that the calculation results obtained from edge servers are accurate. Thus, security, privacy and trust are a priority need for more studies, research, solutions, and mechanisms considering the integration of edge computing and blockchain that has not been investigated thoroughly for VEC computation offloading scenarios. The following, Table 1, shows a qualitative comparison among the reviewed surveys based on some criteria whereas Table 2, shows the comparison between some current studies solution approaches 

**Table 1.** Qualitative comparison among the reviewed surveys.

Reference	Year	Domains					Main objective
		Vehicular network	Edge computing	Computation offloading	Security	Blockchain	
**8**	2021	*				*	Discuss blockchain-based applications in various vehicular networks including edge computing
**26**	2021	*				*	
**27**	2022	*				*	
**28**	2023	*				*	
**29**	2020	*				*	
**30**	2021	*	*			*	
**12**	2021	*	*			*	
**31**	2021	*	*			*	
**32**	2020	*			*	*	Discuss blockchain-based applications in vehicular networks for security objectives
**33**	2020	*			*	*	
**34**	2021	*			*	*	
**35**	2021	*			*	*	
**36**	2022	*			*	*	
**37**	2021	*	*		*	*	
**6**	2021	*			*	*	
**18**	2020	*			*	*	
**38**	2022	*			*	*	
**39**	2018	*	*	*			Concentrate on vehicular computation offloading applications
**40**	2019	*	*	*			
**16**	2020	*	*	*			
**20**	2021	*	*	*	*		
**41**	2020	*	*	*			
**42**	2021	*	*	*			
**43**	2022	*	*	*			
**44**	2021	*	*	*			
**Our survey**	2023	*	*	*	*	*	Focus on vehicular computation offloading security issues and blockchain as a security solution.

**Table 2.** Comparison among different studies consider integrating blockchain into vehicular networks as security solutions.

Reference	Year	Objective	Approach
45	2021	Enable security and privacy for IoV in message dissemination scenarios	Reputation management, incentive distribution mechanisms and voting based consensus for rely selection mechanism
46	2019	Optimize the consensus management	Reputation-based voting mechanism and contract theory
48	2021	Ensure fairness of distributing tasks and data integrity in vehicular edge computing (VEC)	Scoring model, reputation model and incentive mechanisms besides a multi-weight consensus algorithm
49	2018	Focus on information and energy security interactions in electric vehicles cloud and edge computing	Distributed consortium blockchain and inspired data coins and energy coins based on proof of work PoW
50	2018	Focus on secure data sharing in vehicular edge computing (VEC)	Two smart contracts (DSSC) and (ISSC), one for data storage and the other for information sharing and a reputation scheme with three-weight subjective logic (TWSL) model.
51	2021		5G and blockchain, score model without using roadside units (RSUs) and an improved dPoS consensus algorithm.
52	2021		Smart contracts to guarantee a trustworthy environment, PBFT consensus and enhanced reputation-based data management’s security.
53	2019	Aims to secure blockchain-based emergency- driven message (EDM) protocol for 5G enabled VEC.	5G and edge computing, private blockchain to store EDM records.
54	2020	Specifically propose security solutions for computation offloading in edge environments	A reputation blockchain-based model, proof of elapsed time (PoET) consensus, and a voting mechanism.
55	2021		BFT-based proof of stack (PoS) consensus and smart contract, contract-based incentive scheme.
56	2021		dynamic PoW consensus (dPoW) with a checkpoint mechanism and a resource assignment policy in social internet of vehicles (SIoV).
61	2022	Enhance the performance of edge computing with blockchain to enable V2X communications	Multi-chain architecture based on the main chain.
47, 57– 60	2019– 2021	Provide different frameworks for authentication purposes in vehicular network	

## Preliminary background

### VEC overview

Based on edge computing motivations, VEC is a promising technology that appeared to develop ITS and smart city applications. VEC is formed by integrating some technologies, including vehicular networks, smart vehicles, and edge computing. Although VEC has the same concept as MEC in providing computational resources proximate to vehicles and users, VEC needs to deal with the vehicular networks' unique characteristics, such as high mobility ^31^. VEC paradigm comprises two cloud types, vehicular cloud (VC) and Edge, that can be used in an isolated or integrated way through V2V or V2I connections. Edge is composed of a set of edge servers deployed along roads attached to RSUs or BSs. On the other hand, VC is a pool of vehicular computing resources that can be cooperated and offer services on-demand through V2V connections ^16^.

According to the Automotive Market Research Report titled “HOW MANY CARS ARE THERE IN THE WORLD IN 2022?” about 1.446 billion vehicles will be on Earth in 2022. Most of the cars in the world are in Asia, followed by Europe, North America, South America, and Middle East countries come in fifth place ^64^. Nevertheless, vehicle resources tend to be underutilized continuously, whether parked, stopped at traffic lights or in congestion. That means there will be idle resources that can be leveraged by another vehicle with a shortage or busy resources to make services and applications ^31^. Due to the vehicles constrained capacity, vehicles can offload their computation-intensive and time-sensitive tasks to the nearby edge servers or vehicles. These computing platform servers are isolated from the rest of the network and can be in a remote location connected to or disconnected from the data centers of the traditional cloud. They are available to users within the radio access network (RAN) and can operate with little or no Internet connectivity. Therefore, computation offloading can considerably reduce the response time and efficiently alleviate the heavy burden on backhaul networks ^12, 16^.

The primary purpose of building VEC is to allocate resources and services to the vehicles needing additional resources and therefore increasing a vehicle's resources range and extending its computing capability and users' services capacity. Furthermore, it focuses on meeting the execution requirements for vehicular applications, such as lower latency ^31^. Modern vehicles in VEC are supposed to be equipped with powerful communication resources, including processing and storage. A specific example of such powerful vehicles is smart vehicles. Smart or intelligent vehicles can carry out communication processes and manage their actions more appropriately based on monitoring their environment intelligently with the help of several sensors and other embedded units. However, although modern vehicles have such capabilities, they have a limited capacity for computing resources. This limitation usually does not meet all the requested services, which may drive the vehicles to request a server to compute a service, *i.e.,* offloading ^31^.

***Computation offloading processes and architecture-based VEC.*** According to ^42^ and ^41^, Computation offloading processes can be divided into four main steps, task division or partitioning which means offloading can be done for an entire application simultaneously, or the application can be divided at first and then offloaded. Then, in the offloading decisions or scheduling stage, it is decided if the offloading is needed and where it will be offloaded based on available resources and processing policies such as considering the mobility of vehicles. After offloading an application or task to an appropriate edge server, the required computing resources are allocated. Finally, the results of the computation offloading are sent back and received by the requester vehicle with intermittent connectivity in consideration.

**Figure 2.**
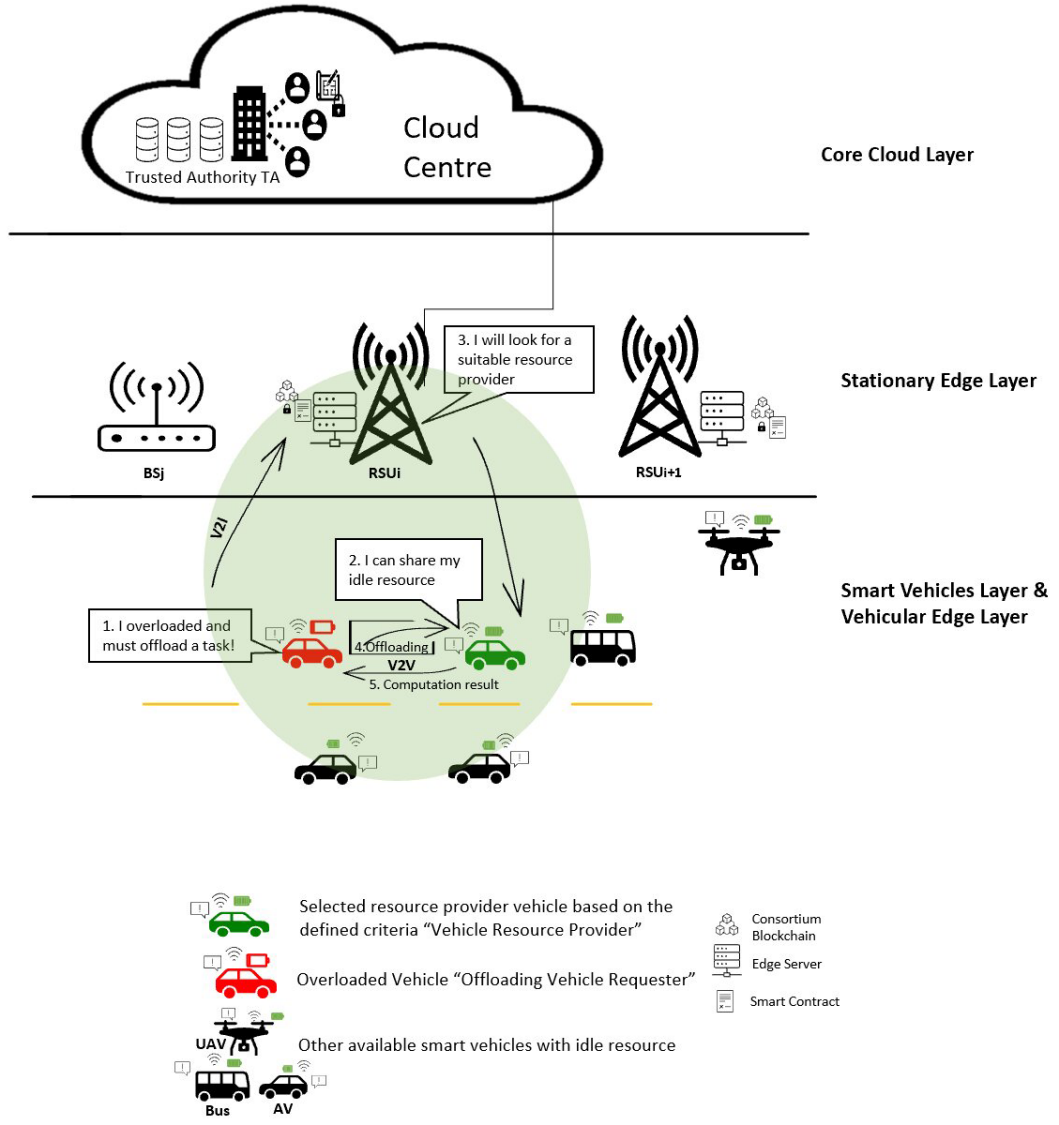
Four-layer VEC architecture and Basic computation offloading processes.

Most VEC architecture in the context of computation offloading is focused on two-layers architecture and others use the three-layers architecture, which includes the core cloud in addition to providing more powerful computation when the lower two layers are insufficient. However, the authors in ^20^ expanded the traditional three-layers architecture into four-layers. This architecture divides the edge layer and differentiates it into stationary and vehicular layers, which works appropriately in the context of VEC. Following Figure 2 illustrates these layers and shows basic computation offloading processes then a brief description of each layer is listed.

**•   Smart vehicle layer**

This layer, the mandatory layer for each VEC architecture, consists of any intelligent autonomous vehicles (AV) types with low resource availability that need to offload tasks. The smart vehicle in this layer can call an offloading requester, a client, or a task vehicle. Furthermore, the vehicle can fully or partially offload tasks or cache data to edge nodes within the range ^20^.

**•   Vehicular edge layer**

All other smart vehicles with idle resources, either a parked vehicle or a moving vehicle belong to this layer. Such a vehicle plays the role of a server, which can be called Vehicle as a Resource (VaaR) or a resource provider vehicle. Buses and unmanned aerial vehicles (UAVs) are vehicles apart from cars that can be considered in computation offloading scenarios ^20^.

**•   Stationary edge layer**

This layer could be RSUs, or BSs, which contain computing resources. The stationary node integrated with the edge server usually handles data distribution, storage, and computation. The proximity of RSUs to both AV and edge servers makes them preferable to serve the requesting vehicle than BSs. However, BSs may still serve the requesting vehicle if no RSU coverage exists. Additionally, the stationary nodes with an edge server have powerful resources which make them further costly than the vehicular node ^20^.

**•   Core cloud layer**

It is a central association of many servers with more stable connections, larger capacity, and higher computation resources. However, according to ^20^, only several works have considered cloud computing platforms in their VEC architecture because involving more layers could increase the complexity of the proposed model. Therefore, it can be assumed that this layer can be optional in developing the computation offloading solutions ^20^.

### Computation offloading overview

The emergence of intelligent and autonomous vehicles and their new complex applications demand extreme computation resources. However, with their available computational resources, the current vehicles are insufficient to run such intensive computation applications. Therefore, other vehicles, edge servers, and remote cloud servers can be exploited to accomplish complex application execution by lending their resources ^16^. 

A technique called computation offloading is necessary to utilize these idle resources and move a task or complete applications to other nodes in the network for performing the execution. The performance and quality of service (QoS) for these applications—and even the entire network—are improved by computation offloading, which also alleviates the computational load on overloaded vehicles and enhances the computing capabilities of the vehicles themselves ^16^. Furthermore, to achieve faster response time, VEC is leveraged by bringing computation closer to vehicles and integrating computing edge servers within BSs or RSUs, and adjacent vehicles ^20^. Intelligent vehicles will significantly be improved as a result of the integration of computation offloading processes, edge computing, and vehicular networks. However, while adopting computation offloading, it is crucial to take into account the highly dynamic environment of vehicular networks and communication reliability issues ^16^.

**Figure 3.**
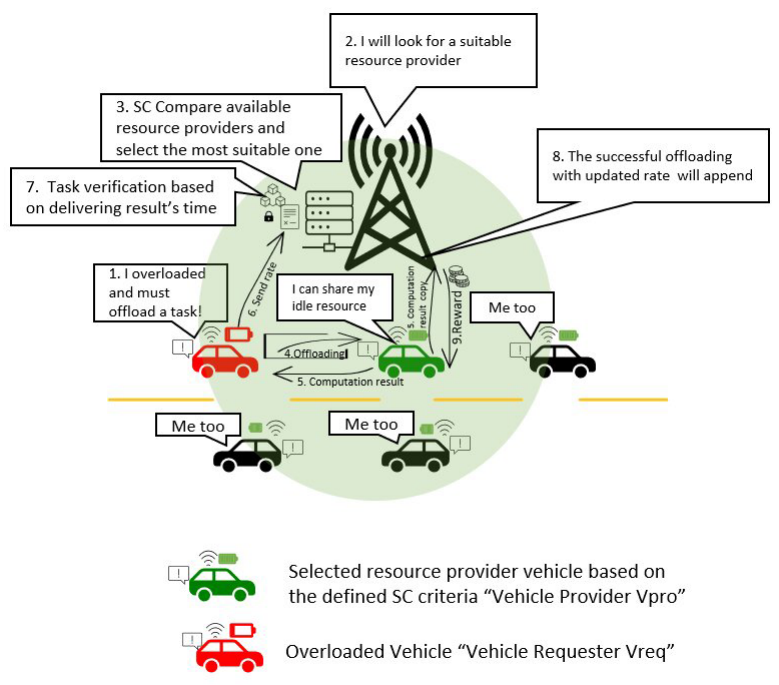
Basic V2V computation offloading scenario.

***Computation offloading definition and categorization.*** It is called computation offloading when a decision is made to migrate intensive computation tasks in mobile devices or vehicles to edge servers. Also, we can refer to this terminology by different names, including task offloading and workload offloading ^42^. In the VEC case, computation offloading refers to transmitting the intensive computational tasks to neighbor edge servers from a vehicle’s on-board unit (OBU) for processing. This transmission supports mitigating the overload of the vehicle and executing the tasks with ultra-low latency ^20, 42^. The following Figure 3. illustrates the basic scenario of computation offloading between two vehicles with the help of RSU in the middle.

Computation offloading can be categorized as static or as dynamic. For example, when defining which application should be migrated and where at the time of installation before execution is called static offloading. On the other hand, computation offloading will be dynamic when the present network statues, mobile devices, and remote server parameters determine which applications should be migrated and where ^16^. Moreover, computation offloading could happen among vehicles and edge nodes within the same server coverage area or through utilizing different server nodes in more than one coverage area. If the vehicles utilize the edge server within its cloudlet for computation offloading, it is called intra-offloading. Whereas, if the vehicles used different edge servers outside the same cloudlets by overhanding tasks, it is called an inter-offloading scheme ^20^. 

***Computation offloading objectives.*** Commonly, computation offloading in VEC considers the optimization of objectives such as QoS, energy, and revenue. For example, it guarantees QoS by minimizing the latency, response time, and message overhead. Also, it optimizes power efficiency by reducing the total energy consumed by computation offloading. Moreover, regarding revenue maximization, an incentive mechanism is employed to increase the willingness of vehicles to participate and share their resources ^20^.

Various works on vehicular computation offloading put forward algorithms and solutions to improve one object or more, *i.e.*, joint optimization. They focus on optimizing response time, energy consumption, financial cost, overload, system utility, incentive, and offloading reliability ^16^. However, based on recent surveys like ^16, 20, 42^, it is noticeable that there is no main objective focus on securing computation offloading which needs more considerable effort besides obtaining appropriate solutions for the vehicular networks’ environment.

***Computation offloading strategies.*** Researchers use different strategies to solve problems and fulfil the computation offloading objectives. These strategies include stochastics, game theory, mathematical programming, metaheuristics, and machine learning. The algorithmic approach that uses machine learning has recently emerged. It uses algorithms with deep learning that can make dissection in the offloading process more efficient. However, it may take more time and consume more computational resources to offer the best results. Yet, algorithms that take less time and consume less computation may not make an efficient decision for the offloading process. Therefore, there is a trade-off between generating an appropriate decision for the computation offloading and time with computational costs ^16^. In addition, there are strategies related to network management that are usually applied on vehicular networks, which are clustering techniques and SDN. SDN architecture brings interactions between applications and devices (real or virtual) and network services closer together, making them programmable to simplify and optimize network operations ^16^.

In short, as to ^42^, computation offloading schemes can be categorized generally based on their strategies and optimization objectives into learning-based, game-based, mobility-based, joint offloading, delay optimization, and energy efficiency.

***Computation offloading applications.*** VEC- based applications are delay-sensitive tasks aimed at efficiently saving drivers' and pedestrians' lives on roads ^42^. Applications in vehicular networks are commonly classified as safety applications and comfort applications. Safety applications are a priority and the most important one due to their responsibility to improve road safety, prevent accidents, and increase the ability to respond appropriately in different situations. Comfort applications are responsible for enhancing the driving pleasure of vehicle occupiers by providing infotainment and entertainment ^16^. Based on ^43^ and ^16^ we can categorize these various applications into two categorizations as follows.

*1. Categorization based on various application areas*

There are various application areas of computation offloading in vehicular networks explained in ^43^ which include, object detection traffic management augmented reality (AR), video analytics, autonomous driving, content distribution and parking assistance. For instance, object detection applications utilize surveillance applications within vehicles’ cameras to monitor traffic which also continuously record real-time photos and videos to identify objects. Traffic management applications can be enhanced by utilizing accident and road blockage information, such as lane change warnings, speed management, and weather detection. Whereas autonomous driving applications aim to provide safe navigation while facing various changes in the surroundings. The process of perception, planning, and control are necessary to fulfil the autonomous driving goal. Parking assistance applications with the help of edge servers can provide parking services in a real-time manner by handling vehicle requests and finding a suitable parking slot. Directing vehicles to available parking increases the effectiveness of traffic and parking management while reducing required time and fuel usage ^43^.

Consequently, such kind of applications must support accuracy processing since they are time sensitive, and these processes require high computational resources either provided by the vehicles’ onboard sensors or from various surrounding servers. Certain kinds of content, like emergency warnings, are time-sensitive and demand low latency ^43^.

*2. Categorization based on processed data type*

Regardless of all the various applications, the processed data type can affect the application performance and computation offloading extremely in the context of transformation and processing time. Therefore, in ^16^ the applications are primarily classified based on the type of data they will process into video, image, audio, and others.

Video related applications such as AR, real-time navigation, and surveillance systems can develop safety applications while online gaming, video streaming, and tourist information can support comfort applications ^16^.

Image Related Applications leverage the installed vehicles’ cameras for safety in situations like using license plates to recognize stolen vehicles or for comfort-related applications such as in social networks ^16^.

Audio related applications are commonly based on human voice recognition. One example of audio safety applications is emergency calls, while voice chat and guided tours are examples of comfort applications ^16^.

Consequently, the massive amount of data and energy generated by processing such video-related applications is a challenge that affects computation offloading performance. Image and audio-related applications consume lower processing time and computation costs than video applications since their size is basically smaller. In addition, there is an exciting development of other applications type in the vehicular environment that can improve performance through computation offloading. Thus, regardless of which variety of applications, it is crucial that computation offloading maintain satisfactory performance ^16^.

***Computation offloading challenges.*** There are various challenges facing computation offloading in VEC in regard to network, mobility, security and privacy, incentive, and algorithm ^16^ that need consideration to achieve advance performance in computation offloading. The following list discusses these issues regarding each aspect, as discussed in ^16, 20^.

*1. Network and mobility*

Extreme congestion network in the vehicular network affects the success of the computation offloading due to:

•  Edge node density at road junctions could disrupt the offloading if there is a high density. It may face extensive offloading requests where the edge node number is low, and computation offloading requests are extremely occurring.

•  Signal attenuation issues due to obstacles increase the delay, specifically if a multi-hop transmission is used.

•  The heterogeneity of the technologies, which are utilized to improve bandwidth, requires efficient management. For example, modern technology like 5G needs more studies to adapt it to vehicular networks efficiently.

Moreover, fast speed and dynamical vehicular networks lead to short-lived connections, failed or intermittent wireless communication, moving outside a communication range, and massive packet loss. Predicting the staying period of nodes within the computation offloading range can considerably mitigate the mobility issues consequences. 

*2. Security related issues*

Issues related to security can be separated into four classifications as follows.

•  Data encryption: sensitive data can be sent to a malicious server or a malicious node. Even though adding encryption and authentication mechanisms can ensure confidentiality, it adds more overhead in computation power and time processing, affecting the efficiency of offloading, QoE, and system scalability.

•  Trust: the vehicular environment has an excessive number of untrusty entities that do not know each other. Therefore, potential servers likely refuse to deal with offloading requests from the unknown requester, resulting in failed computation offloading. 

•  Security: Due to a lack of security strategies, uploading tasks containing viruses or harmful configurations by malicious nodes is likely. Moreover, different security attacks can affect the computation offloading processes like distributed denial-of-service (DDoS) attacks.

•  Privacy: there is a potential threat of privacy leakage of offloading requests. Thus, trust management, data isolation, and anonymous authentication can improve privacy.

*3. Incentive*

Computation offloading is based on sharing and lending resources that need cooperation between all entities in vehicular networks. Performing tasks with another vehicle’s resources mitigate the requester's workload and cost the provider vehicle more energy. Therefore, the chance of selfish nodes using the borrowed resources all the time is increased, and the willingness to lend resources is decreased. As a result, building an incentive mechanism and considering different situations to motivate nodes in order to cooperate and reward the cooperated nodes need additional consideration.

*4. Algorithm*

Computation offloading is a set of mechanisms and algorithms that work together to accomplish the offloading goals of alleviating an intensive load of a vehicle and improving its performance by transmitting complex tasks to an appropriate edge server. Algorithms-related challenges of vehicular computation offloading are some of the biggest challenges to consider, such as load balancing and partitioning, failure handling and unstable connections, discovery and resource request and task distribution or task scheduling.

### Communication standards and technologies

Generally, three types of communications facilitate offloading techniques including V2V, V2I, and V2X. Offloading through V2V can happen directly among compact vehicles where there is no need for stationary infrastructure support. BSs in the most direct communications can provide signaling control. Nevertheless, vehicles’ limited resources, mobility, locations, and the shared period should be determined and addressed ^39^.

Moreover, the stationary infrastructure, including RSUs and BSs, is necessary when offloading through V2I communications to provide connectivity and services. However, fixed edges should address limited coverage, installation cost, and network maintenance issues. However, V2X communications combine V2V and V2I communications and overcome their disadvantages where a vehicle can connect to either neighbor vehicles or local stationary infrastructure depending on the network conditions ^39^. Precisely, it is essential to decide the communication approach in vehicular computation offloading regarding communication technologies, client and server, type, and wireless hop numbers as following explanation.

***Communication technologies.*** Different communication technologies are enabled in vehicular networks, which mainly include Wireless Access in Vehicular Environment (WAVE) and cellular networks. WAVE is a group of protocols standardized by the IEEE for vehicular network communication, and one of them is called dedicated short-range communication (DSRC) ^16^. These protocols provide direct connectivity among vehicles or infrastructures through V2V and V2I whereas cellular networks provide higher capacity and higher-speed communication. The two most cellular networks used in computation offloading in the vehicular network are 4G and 5G. 5G networks extend the communication range and enhance communication performance ^16^. Based on research conducted by ^16^, most works use WAVE to enable computation offloading in vehicular networks. Few uses cellular networks, while others use both technologies simultaneously, such as WAVE and 5G.

***Client and server.*** The client means the requester node has overloaded computation and must send an offloading request. It could be a vehicle, infrastructure, or pedestrians; however, the vehicle most commonly acts as a client. On the other hand, Server means the node that receives the offloading request from a client and processes it. Various nodes can act as servers, including vehicular clouds or a group of vehicles, edge servers equipped with RSUs or BSs, and traditional cloud. Determining whether one server type or more than one type to use is based on the application requirements regarding latency and computing power. It is worth mentioning that a vital trade-off between latency and computational power should be considered when deciding where to perform the computation offloading ^16^.

***Communication type and wireless hop numbers.*** The communication between clients and servers during the offloading processes can be done directly through one-hop wireless or multi-hop, *i.e.*, relay nodes. Each way has its usefulness based on the required objectives. Avoiding unnecessary delays and minimizing the probability of offloading failure can be reached using one hop, which is the most common approach. Conversely, multi-hop increases the latency and the offloading failure rate, although it increases the communication range and provides powerful resources that are distantly placed ^16^. 

### Blockchain overview

A public ledger that keeps data in a sequence of blocks in a decentralized, distributed approach is referred to as a blockchain. Blockchain was originally suggested by Nakamoto in 2008 for supporting Bitcoin, a virtual cryptocurrency. However, blockchain has been growing, and its idea has been considered in different fields, more than just the financial sector ^23^. 

Blockchain is regarded as a promising technology for the next generation of the internet era, including IoT, reputation systems, and security services. It enables the transformation of digital assets in a peer-to-peer network without requiring any intermediate authority to confirm and approve transactions. In addition, blockchain has beneficial features that secure transactions and make them tamper-proof, such as immutability and auditability. Furthermore, the digital signature, cryptographic hash, consensus algorithm, and smart contracts are applied to facilitate the decentralization environment in the blockchain. Shortly, we can describe blockchain as a distributed ledger technology (DLT), which allows trusted tasks without the need for a trusted entity in an untrusted environment ^8, 23, 24^. Therefore, blockchain considers a trust-less system, meaning that does not require trust to do transactions like the traditional system. Instead, it provides confidence through its unique distributed nature that depends on committing transactions after propagating a distributed consensus mechanism. Blockchain commonly uses a secure hash algorithm known as SHA-256 and asymmetric cryptography called elliptic curve digital signature algorithm (ECDSA). Therefore, the consensus mechanisms and the cryptographic hash function, besides digital signature authentication, can address security issues ^8, 23, 24^.

Generally, we can consider that blockchain is based on five techniques, data Structure and management, cryptography, consensus, incentive mechanisms, and smart contract which will be explained in the following sections after defining the blockchain architecture and types.

***Blockchain architecture.*** The decentralized and distributed blockchain architecture is implemented by several distributed nodes, each containing a replica of the transaction records. These various transaction records are called blocks. Miners involved in the network need to validate these transactions to commit the block and add it to the blockchain. In view of the immutability of the blockchain and the connection between the blocks in the chain by a reference hash of a parent block, it is not possible to amend or delete previous transactions. The initial block with no previous blocks is called the genesis block, while all previous blocks are called parent blocks ^8^.

**Figure 4.**
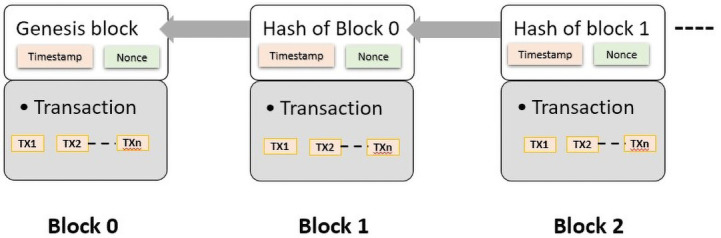
Blockchain structure.

There are two parts to each block, a block header, and a block body. The block header contains all the metadata about the block, such as its version, previous hash, timestamp, and nonce whereas the block body holds transactions and transaction counters. There is a limit on the maximum number of transactions that can be stored in a block based on the transaction and block size ^23, 24^. The following Figure 4 illustrates the general blockchain structure.

***Blockchain types.*** Blockchain is categorized into public, private, and consortium blockchains according to the network access restrictions and consensus participants' identification. A public blockchain is permissionless, entirely decentralized that enables all nodes to enter the network and joins the consensus processes. Therefore, all nodes have the read/write permission of the transaction records. The famous Bitcoin and Ethereum platforms are operated based on the idea of the public blockchain ^23^. By contrast, private and consortium blockchains are permission networks. That means only nominated authorized nodes can participate in the consensus, and the network access is restricted based on some policies. However, the consortium, unlike the private blockchain, which is regarded as being totally centralized, is only partially centralized and not run by a single authority ^23, 24^. Table 3 shows a brief comparison between these three types of blockchain.

**Table 3.** Blockchain types.

	Public	Private	Consortium
network access	permissionless	permission	permission
consensus participants	all nodes have the permission	restricted nominated authorized nodes	restricted nominated authorized nodes
Structure	entirely decentralized	totally centralized	partially centralized

The public blockchain provides low efficiency due to its openness and available joining nodes, which lead to high latency and low throughput ^23, 24^. These issues prevent using public blockchains for time and energy-sensitive applications such as intelligent transportation. On the other hand, the consortium is in the middle between the public and private blockchains, and it is considered partially centralized. That means fewer selected validators and restriction access can improve performance in providing acceptable latency, energy, and throughput rates ^27^.

The most appropriate blockchain type for vehicular networks is controversial in the field. In real vehicular network scenarios, conventional public blockchain-based solutions are unsuitable, according to various researchers. Their recommendation is to use consortium blockchain to enhance vehicular networks' security, privacy, and trust. Consortium blockchain-based solutions offer benefits such as restricted access, high efficiency, and scalability. Additionally, the private blockchain is sometimes thought to be a satisfactory approach to cope with challenges related to security, privacy, and trust in vehicular networks ^37^.

***Blockchain basic techniques.*** Generally, we can consider that blockchain is based on five techniques, data Structure and management, cryptography, consensus, incentive mechanisms, and smart contract which will be explained in the following sub-sections.

*Data structure and management*

Two different structures can be used in the blockchain network. First is the common linear way that is defined in the previous architecture section. The second is the non-linear structure called a directed acyclic graph (DAG). In this structure, every block is connected with its previous multiple blocks ^8^. Blockchain-based DAG, like IOTA, can process transactions in parallel, which means that in DAG, more than one block can be processed simultaneously, which increases the throughput ^38^.

There are three techniques for data management include on-chain, off-chain, and side-chain. In the on-chain approach, the common one, the content of all blocks is stored and processed within the blockchain network, which is accessible to all nodes. In contrast, some data are stored and processed externally outside the blockchain network to accelerate the implementation in the case of off-chain management ^8^. Whereas the sidechain is a fully independent blockchain linked to the main chain. It utilizes the idea of parallelism ^8^. Moreover, according to ^65^, the side-chain approach can reduce the transactions number over the network and the transaction throughput can increase by adding more sidechains.

In addition, the “sharding” technique is worth mentioning, which has similarities with sidechain techniques. Although it can help increase throughput and enhance scalability, it faces the same security challenges as sidechain networks. However, it has the same idea behind sidechain networks, which is based on partitioning the leading network into parts or shards. Each shard is controlled and managed by a specific committee instead of the entire network. Inspired by the sharding approach, Aspen and Elastico are proposed, and they have different transaction methods depending on the checkpoint blocks and epochs mechanism, respectively ^65^.

*Cryptography techniques*

Cryptographic techniques are leveraged to ensure security, privacy, and anonymity in blockchain technology, including hash functions and digital signatures. For example, as explained in the blockchain architecture, each block has the hash value of its parent block, making the chain immutable. Also, the Merkle hash tree summarizes all previous transactions in a block which helps to reduce the storage overhead and speed up the integrity check processes. Therefore, the hash values make the blockchain tamper-proof and ensure its integrity ^8^.

To guarantee authentication and non-repudiation, a digital signature like ECDSA is commonly used, while some blockchains use signatures such as ring signature to hide the signer’s identity. Besides all that, the blockchain utilizes zero-knowledge proof to enhance privacy ^8^.

*Smart contract*

The concept of smart contracts, which was proposed a long time ago, can be integrated into blockchain platforms. The smart contracts are auto scripts established in the blockchain that will be executed automatically when triggered or met some predefined rules or conditions. Leveraging smart contracts aims to support and develop autonomous systems, which do not rely on any third trusty entity to provide efficient services. Therefore, it will help reduce processing time and administrative costs and advance many industrial sectors. Utilizing Smart contracts will develop blockchain functionality better than just a simple distributed ledger. The blockchain-based contract will inherit the blockchain features regarding security and traceability, and inalterability ^8, 24, 25, 38^.

Utilizing smart contracts can optimize both demand and supply for data trading or computing resource sharing and task scheduling for VEC applications. They can also be utilized for security reasons, such as when certain nodes require authorization to execute ^38^. However, smart contract vulnerabilities and defects can lead to ruinous damage to the system. Thus, considering attacks analysis when deploying smart contracts is a critical demand ^8, 24, 25^.

*Incentive mechanism*

Modelling an efficient incentive mechanism is required to encourage constructive cooperation with all nodes in the vehicular network, mitigate destructive behavior, and punish malicious nodes. There are commonly two strategies to build incentive models: price-based and reputation-based. Price-based mechanisms depend on trading ideas in addition to using virtual credits to exchange information. The reputation-based mechanisms rely on measuring trustworthiness to build cooperation. Based on a set reputation threshold, node behavior can be distinguished as trust or malicious. Usually, malicious nodes are punished by applying a punishment scheme. Based on a game-theoretical analysis, integrating both strategies is recommended because combining the advantages of both strategies leads to higher effectiveness in encouraging positive cooperation and detecting malicious nodes ^66^. 

*Consensus mechanisms*

Consensus is used to build trust in the blockchain network, which is decentralized and not dependent on any trusted entity. Consensus helps in making an agreement among nodes in a trust-less environment in order to validate a task and transparently commit a block. Each mechanism has unique rules and algorithms to validate and create blocks ^8^. The process of gathering, verifying, and packing transactions into a block based on the type of blockchain and consensus mechanism is called the mining process, and all participating nodes in this process are called miners. Some of the notable consensus mechanisms are proof of work (PoW), proof of stake (PoS), delegated poof of stake (DPoS), practical byzantine fault tolerance (PBFT), and Tendermint ^23, 38^.

• **PoW**, which the Bitcoin network uses, is a proof-based consensus algorithm. Determining which node has the right to attach a new block to the chain is based on sufficient effort proof. In PoW, nodes are competing trying to answer a complex puzzle and guess the appropriate nonce to win the opportunity to append the new block after broadcasting it with the nonce in the network to every node to verify it. The node that found the solution first will receive a reward. However, miners demand consuming high computational resources during this mining process ^23, 38^. 

• **PoS** can be an efficient energy replacement for PoW since miners do not need to solve the mathematical puzzle and consume vast computing resources. Instead, they provide proof of possession a sufficient stake in getting the opportunity of validating and creating a new block. Therefore, choosing the validators depending on the amount of the participant's stake can mitigate the possibilities of malicious attacks and save more energy. However, the chance of an unfair decision arises because the wealthiest nodes have the highest probability of being selected as validators ^23, 38^.

• **DPoS** is not a proof-based consensus but an elective-based consensus, which depends on voting processes. Each node with a stake is able to delegate the validation of a transaction to an alternative node by voting. The elected nodes or called witnesses take turns voting and validating blocks. Because the participants' number is fewer than in PoS, it accelerates block generation and validation. However, it tends to be more centralized ^23, 38^. 

• **PBFT** is one example of BFT mechanisms. BFT means the ability to make consensus safely among nodes communicated in a distributed network despite malicious nodes' presence. PBFT consensus depends on a set of trusted nodes assuming the presence of dishonest or faulty nodes. There are backup nodes and a leader responsible for ordering a transaction, and some rules select it. Then, based on the majority votes, decisions are made. Generally, the process is done in 3 phases, including preprepared, prepared, and commit. Then, votes from over 2/3 of nodes must be received to move from one step to the next. That means a consensus in PBFT can be done with the existence of 1/3 of malicious nodes effectively ^23, 38^. 

• **Tendermint** is based on byzantine consensus algorithms. Its processes are similar to the PBFT consensus. There is a selected proposer to broadcast a block and then follow the same three processing steps in PBFT. However, nodes in Tendermint become validators if they lock coins, while they will get punished if they are found dishonest ^23^. 

Discussing and covering various consensus mechanisms requires writing a specific survey because many consensus algorithms have appeared with different goals and applications. Thus, each consensus has its advantages and disadvantages. Moreover, some properties can differentiate these consensuses, node identity management, energy-saving, and adversary power tolerance. For example, PoW and PoS are commonly appropriate for the public blockchain, while consortium or private blockchains prefer PBFT, Tendermint, DPOS, and Ripple ^23^. Regardless, the ^65^ survey investigates and discusses the consensus algorithms and mining strategies in depth. Moreover ^23^, and ^52^ discuss some notable consensuses and provide a comparison table between these consensuses.

## Security in vehicular networks and blockchain as a security solution 

### Security-related issues in vehicular networks generally

This section purposes a perception of the security in vehicular networks generally, and the next sub-section discusses the security aspects related to computation offloading applications specifically, which additionally will inherit all the security-related issues faced the vehicular networks. It is worth mentioning that when we say security issues, it usually includes all three aspects of security, privacy, and trust.

***Security requirements in vehicular networks.*** Implementing efficient security methods is required to ensure satisfying the ITS goals and the operation of the vehicular networks appropriately. Therefore, five essential security requirements must be considered when developing a good security solution: availability, confidentiality, authenticity, data integrity and nonrepudiation ^5, 37^.

•  Availability guarantees that the vehicular network and its related applications provide continuous operation in the existence of malicious or faulty statuses. The performance of vehicular communications will be deteriorated when there is a lack of available services ^5, 37^.

•  Confidentiality guarantees that only the authorized recipient has permission to access confidential information. Any other nodes cannot access or understand this information related to each network entity. Confidentiality can be provided by using cryptographic solutions ^5, 37^.

•  Authenticity protects vehicular networks against malicious nodes. It is known to be the initial line of defense against different vehicular network attacks ^5, 37^. Nodes in vehicular network topology frequently join and leave since it is a dynamic network, thus authentication has a critical role in such topology ^38^. However, computational and communication overhead is a challenge in regard to authentication solutions besides other challenges, such as suitable bandwidth utilization, scalability, and decreased response time ^37^.

•  Integrity protects the original content of a message from any alteration through transmission. This means integrity helps in avoiding unauthorized data creation, destruction or modification ^5, 37^.

•  Nonrepudiation, in case of dispute, makes sure that the sender and receiver of messages cannot deny the delivering and accepting broadcasting messages ^5, 37^.

**Figure 5.**
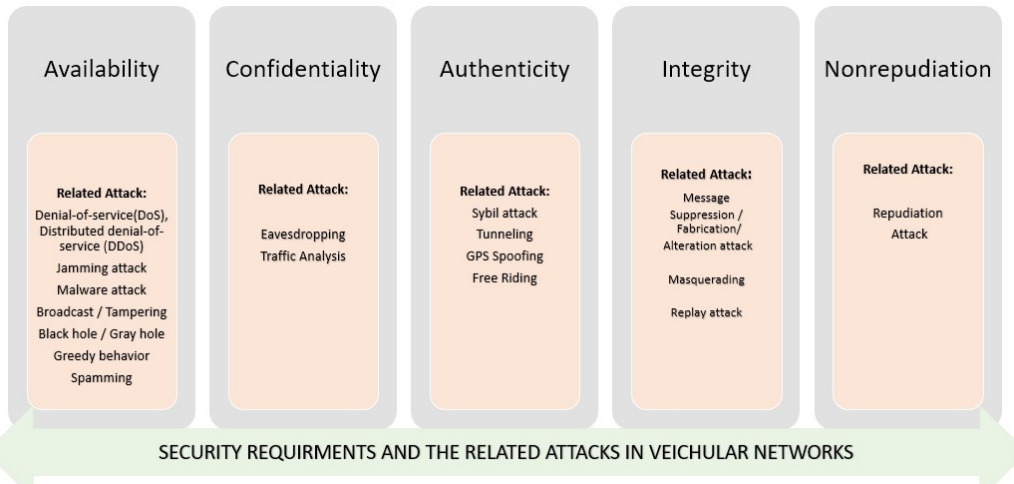
Illustrates attacks corresponding with each security requirement.

In addition, each security requirement has its related attacks, which ^5, 37^ provide a good explanation and references for each attack. In Figure 5, these attacks are illustrated based on ^5, 37^.

***Privacy preservation in vehicular networks.*** The vehicles’ privacy is as important as their security, which needs to be considered. According to the basic definition of privacy, individuals have complete control of their private data and have the authority to decide which nodes can access it and what information they can share. In the vehicular environment, privacy maintains both types, the privacy of vehicles and the privacy of their location. Thus, only dedicated nodes in the network have access to and control over the data in relation to the identity of the vehicle and as well as its location. The standard way for preserving privacy is anonymity authentication, which keeps the real identity unconcealed by utilizing a digital pseudonym, *i.e.*, a bit string ^5, 37^.

Personal information is protected from fraudulent users or attacks via authentication mechanisms, which ensure the distinguishing of adverse nodes, fake messages, and forbidden entities by using various cryptographic techniques. The various utilized cryptographic mechanisms categorize anonymous authentication into symmetric cryptography, public-key infrastructure, identity-based signature, certificate-less signature, and group signature. Privacy preservation techniques based on pseudonyms can be effective and reliable. Nevertheless, even if the broadcasted messages are completely anonymous, attacks like tracking attackers, which might alter the route of the intended vehicle, cannot be prevented by anonymity alone ^5, 37^. A whole section in ^5^ and ^37^ elaborates on these five cryptographic mechanisms. Moreover, in ^5^, the method of a tracking attack is described, and various location privacy protection techniques are discussed as a supplement to anonymous authentication.

***Trust management in vehicular networks.*** One of the vital challenges facing vehicular networks is trust management. How a vehicle can trust its peers and its broadcasting messages is determined by trust management. To guarantee that messages are transmitted by authorized vehicles, several authentication techniques are both considered. An authorized vehicle may distribute revised messages and behave maliciously, therefore, building confidence among verified cars is a critical problem. Malicious messages, such as fake alerts, degrade the effectiveness of vehicular communication systems and threaten people's lives and as a result cause accidents ^5, 37^. It is essential to evaluate the message's credibility and respond accordingly. Each vehicle in the vehicular network must therefore be able to disclose untruthful vehicles and the fraudulent messages they have transmitted. There are three widespread trust management approaches including models of trust that are entity-centric, data-centric, and combination trust models ^5, 37^.

- **Entity-centric trust models** provide efficient vehicle trustworthiness decisions based on the opinions of neighboring vehicles, *i.e.*, reputation system. However, high mobility in the vehicular network makes information collection and reliable reputation calculation difficult. Another important issue that needs to be taken into consideration is establishing a technique to guarantee the reliability of the reputation system itself.
- **Data‑centric trust models** evaluate the trustworthiness of received messages by utilizing the collected Information from neighboring vehicles or RSUs. However, latency, data sparsity, and data redundancy are the main challenges facing this kind of model.
- **Combined trust models** focus on validating the legitimacy of the vehicles and received data. As a result, such models can benefit from entity-centric and data-centric trust management techniques ^5, 37^.

However, the qualities of desirable trust management in the vehicular network can determine each model's effectiveness. A variety of third-party-based trust management strategies, which depend on centralized servers and a centralized reputation system, have been suggested. These mechanisms are inadequate for vehicular networks because of the challenges in maintaining and recovering from failure in such a dynamic network. Additionally, interactions between two vehicles typically only occur once, and even then, there is no assurance that they will do so again. As a result, relying on centralized mechanisms like a trusted third party (TTP) to develop lasting connections is untenable. Decentralized trust techniques are therefore better suited for vehicle networks because they do not totally rely on the static infrastructure ^5, 37^. Several significant challenges are presented for trust management because of the unique characteristics of vehicular networks. Therefore, to design an appropriate trust management model for vehicular networks, five properties should be considered, decentralization, real-time constraint, information sparsity, scalability, privacy and robustness ^5^.

### Security-related aspects in computation offloading

Data security at edge nodes is a critical concern, despite the fact that edge computing offers new possibilities to improve mobile device services and applications. Several applications based on smart cities employ edge nodes for analytics in real-time with high throughput besides the sensitive data produced by this variety of applications, including connected vehicles ^21^. Thus, maintaining these data's security, privacy, confidentiality, and integrity is strictly demanded. Several attacks can be launched against the edge nodes by hackers to compromise security and sensitive data or deny providing services to the requesters. These attacks include but are not limited to DDoS, hijacking, ripple, byzantine, and injection attacks ^21^.

Although the distributed edge computing topology has many advantages, managing its security creates a significant challenge. During message transmissions, for example, attackers can launch such as jamming, and sniffer attacks to disrupt communications by overloading the communications infrastructure or monitoring data movement ^19^. Furthermore, the collaboration between heterogeneous nodes and edge servers as well as the movement of tasks between global and local scales will both present potential malicious behaviors ^67^. Although it is considered challenging due to the high mobility and openness of the environment, trustworthy and validated configurations are required ^19^.

Furthermore, maintaining security and privacy in uploading computational tasks to edge computation nodes is a serious security challenge in an edge computing network. Guarantee offloading safety in a VEC environment can be reached by verifying two main criteria, which are “the reputation of a vehicle ( *i.e.,* trustworthiness) and the behavior of the content ( *i.e.,* anomalousness)” according to ^20^. The reputation of vehicles helps to determine if the vehicles can be trusted before any offloading processing occurs, whether the vehicles are offering the offloading, *i.e.*, server vehicle or requesting the offloading, *i.e.*, requester vehicles. Moreover, the content's anomalousness helps validate a task before migrating it to an edge node ^20^. Based on the research performed by ^20^, the present security schemes involving offloading in VEC have proposed different solutions to optimize security, prevent malicious offloading and prevent attacks such as packet drop, jamming and eavesdropping, besides other optimization utilities including overhead and delay. They used various approaches, such as vehicle rating or trustworthiness with neighboring vehicles’ recommendations, lightweight authentication, and cryptographic algorithms.

Another significant issue is that obtaining fully trusted and correct processing results from edge nodes may not be guaranteed. To assure the reliability of the results, it is important to investigate the accuracy of edge computation. For instance, it's possible that the powerful nodes performing computation offloading are unreliable, in which case the results must be returned with proof. The requester vehicle will be able to confirm the result and the reliability of the computations in this manner. Designing effective verifiable computing mechanisms will therefore be one of the primary factors in the study of VEC applications for verifying the accuracy of the computational tasks carried out by edge nodes ^8, 19^.

Consequently, it is evident that security concerns like securing control at the edge, data storage, computation, and network may require novel developments for adjusting the decentralization, coordination, heterogeneity, and mobility of the edge computing environment. Taking into account how to avoid the excessive encryption overheads that affect the combination of scalability with security in such large edge numbers is also required ^19^. One method for meeting these security needs in edge computing is blockchain technology. Compared to more conventional security measures, it can provide computation offloading in edge computing with higher security standards. This is due to its unique features, including decentralization, immutability, and traceability ^21, 62^. The following sub-section explains that and discusses leveraging blockchain technology as an enhanced security service.

The continuous development of vehicular applications is expected to keep the number of intelligent vehicles growing. Thus, it produces a massive quantity of data to exchange and a significant network traffic load to manage. Besides that, applying traditional storage and management based on the cloud directly creates substantial difficulties in vehicular networks due to the high movement, low latency, complex context, and discrepancy features of vehicular networks. Additionally, ensuring robust interoperability and compatibility among all heterogenic entities in vehicular networks is also tricky ^8^. Therefore, the information transmission and storage infrastructure in such networks must be decentralized, distributed, interoperable, flexible, and scalable to satisfy the future progress of vehicular networks and fully realize the promise of ITS. The decentralized and distributed platform is, however, fundamentally open to cyber-attacks. Therefore, it is crucial to guarantee the security, confidentiality, and reliability of vehicular networks. Accordingly, blockchain technology has already provided enormous prospects in several IoV applications with the help of efficient cryptography approaches and edge computing processes ^8^. Consequently, it has become standard practice to incorporate edge computing and blockchain into a single solution. By integrating blockchain into the edge computing network, the system's network security, data integrity, and computation validity can all be significantly increased ^19, 68^. Furthermore, the integration provides reliable access and management of the network, storage, and computation over many distributed edge nodes. Edge computing, on the other hand, provides the system with an enormous quantity of computing and storage resources that are allocated at the network edge. Thus, the power-constrained devices effectively transfer the mining computation and blockchain storage to the robust edge node. Moreover, it is important to note that off-chain storage and off-chain computation at the edges make storage and computation on the blockchain scalable. Adding blockchain to edge computing applications enhances security, privacy, and autonomous resource management ^19^.

### Blockchain as a distributed security solution

The ability to protect transactions stored in a shared distributed ledger is one of the key success factors of blockchain ^8^. Generally, the distributed nature of the database has resistance to malicious attacks compared with a centralized one. The distributed ledger is secured *via* a verification process that blockchain users can employ. It is gained protection through supporting different levels of encryption, such as the generation of private and public keys and validation of hash value ^8, 69^. Furthermore, in the blockchain architecture, the records in such distributed ledger are replicated throughout the various disseminated nodes, which facilitates addressing the single point of failure problem. Additionally, such a structure of connecting records in a chain and replicating the content makes alteration attempts almost impossible for any record or block already validated by the participating entities and appended to the chain. Consequently, in such a decentralized network, these untrusted nodes can establish trust among each other ^8^. Decentralization, immutability, and traceability of blockchain technology are some of the security features that make it possible for security services to be provided naturally ^21^.

***Motivations to integrate blockchain.*** The motivation to incorporate blockchain technology in technology such as edge computing and IoV, *i.e.*, VEC, and computation offloading are discussed as following besides the explained motivations for using blockchain for security services, such as access control, in computation offloading, which enabled by its unique properties over the conventional security solutions ^8, 19, 21, 38, 62^.

*Decentralization*

In addition to more distributed independent elements like RSUs, vehicles, and people, the decentralized IoV network can offer distributed data management and storage. This will result in a simplification of the operational fundamentals of the current IoV network, which primarily relies on the central decision system. It will additionally improve consumer experiences for vehicle services and reduce operational costs. In particular, a network such as VEC emphasizes the complementary combination of both the decentralization blockchain and edge computing technology that will give an enhanced framework for recording and validating blockchain transactions. Regarding the offloading mechanisms, the offloading and storage of data in the P2P network will be facilitated without depending on a single centralized authority. This guarantees immediate access to information and greatly improves data security for mobile users. Additionally, mobile offloading helps to implement strong access control with good data integrity and system security.

*Distribution*

The ability to effectively accommodate the massive number of diverse nodes allocated or moving between distinct edges can create a distributed control of several nodes along edges and transparently protect the reliability, consistency, and validity of the data and rules with the assistance of the mining process and data replication on multiple nodes. Distribution is able to protect against security threats like an interruption, single-point-of-failure, and availability attacks by synchronizing and replicating blockchain across all peer nodes associated with the network. As a result, even if several nodes become compromised, the services remain operational without any disruption.

*Peer-to-peer (P2P)*

Service requesters and providers can create direct communications with one another with the cooperation of P2P trading, sharing, and interactions among multiple entities. For vehicular network applications such as securing resources sharing and data exchanging between vehicles and RSUs, the P2P capability is highly helpful. Also, Low-latency applications are achieved due to the direct communication between P2P network participants. Low latency response is supported by blockchain application with edge computing, which is scientifically essential for the majority of vehicular applications. Aside from the blockchain's pseudonymous nature, which enables P2P communication without revealing the metadata (the source, the destination, and the content) to anyone, there is a possibility of achieving complete privacy via implementing the management of granting access and control permission of any nods' data without any third parties. Accordingly, it helps to maintain preserved data privacy and security.

*Privacy preservation*

It guarantees against unauthorized observers and prevents the public or violent parties from learning the private information of the participating nodes and drivers. It can be satisfied through ensuring that anonymity protects the nodes' personal information, prevents unauthorized individuals from tracing a node's activity, or "un-traceability," and is typically achieved by utilizing pseudonyms and cryptographic procedures, which reduces the risk of tracking attacks. Additionally, un-link-ability features make sure that attackers cannot link messages received from the same sender, making it difficult to establish that they were delivered to the same recipient.

*Immutability and traceability*

The block creation as a chain connecting with each other through the hash values provides high immutability for vehicular network applications that prevents data tampering and helps with accurate auditing. In industrial applications like smart transportation, traceability can be used to guarantee the accuracy of transactions. Also, utilizing traceability in resource management allows clients and service providers to verify the service level arrangement properly. Traceability via conditional privacy or conditional link-ability by linking a vehicle’s pseudonym to its real identity to enable a government agency or trusted third party to reveal the vehicle's identity if it is found to be malicious and prevent a “double claim” attack.

*Non-repudiation*

It can be met by ensuring the malicious entity is unable to pretend to be another entity in the network, *i.e.*, a masquerade attack because a transmitting node signs all transmitted messages via its anonymous public key. The message is timestamped, which is essential for tracing the sequence of events and message specifics in investigating agencies in the event of an accident, so the node cannot assert that the message was replied. Using secure positioning technologies has made it impossible for vehicles to be dishonest about their location.

*Smart contract*

While making decisions without the contribution of any trusted entities, the smart contract plays a vital part in addressing trust issues. It also makes it possible to deploy and enforce scripts or rules that have already been established. These scripts aid in creating a system that is autonomous and automated. Smart contracts enable nodes engaged in resource borrowing and lending to dynamically coordinate. By automatically executing an on-demand resource methodology for the requester node, it merely facilitates the utilization of resources on demand. They dramatically lower operational expenses by enabling resource consumption in edge computing with trustworthy, autonomous, and effective executions. Smart contracts, for instance, allow the automatic authorization of nodes and separate honest nodes from adversaries. Additionally, it seeks to stop potentially harmful offloading practices and threats to compute resources. Thus, trustworthy access control of the offloading system can be achieved, and the system's data reliability and offloading authenticity can be significantly enhanced.

*Consensus*

Depending on the blockchain type and consensus method, the process of gathering, verifying, and compiling transactions into a block is known as mining, and the nodes involved in this process are known as miners ^23, 38^. Blockchain may build significant confidence among untrusted diverse entities in vehicle networks with the support of the strength of innovative consensus techniques. The consensus process ensures the reliability and transparency of all data transported through vehicle networks and edge entities built on blockchain technology. Consensus is used to build trust in the blockchain network, which is decentralized and does not depend on any trusted entity. Consensus also helps in making an agreement among nodes in a trust-less environment in order to validate a task and transparently commit a block. The validation and creation of blocks differ based on the distinctive rules and algorithms of each consensus mechanism.

*Cryptographic*

The increased security and privacy for vehicle networks are highlighted by the dependence on contemporary cryptographic approaches to address typical security and privacy challenges. Hash functions and digital signatures are two examples of the cryptographic methods used in blockchain technology to guarantee security, privacy, and anonymity. For instance, each block contains the hash value of its parent block making the chain immutable. The Merkle hash tree's ability to summarize all previous transactions in a block also allows it to be utilized to reduce storage requirements and expedite integrity check procedures. As a result, the hash values protect the integrity of the blockchain and prevent tampering.

***Possible applications of using blockchain as a security solution in vehicular applications.*** Edge computing and modern cryptographic techniques have already presented great opportunities in several vehicular network applications ^8^, and computation offloading is one of them. To give a comprehensive view of vehicular applications using blockchain-based contexts for security purposes, the applications are divided into two categorizations based on the application scenarios to be considered and the security features required ^38^. It is worth mentioning that the following applications can be possible applications that utilize the same idea of the BEoV framework to provide various security services, which will be explained later.

*Categorization based on application scenarios*

Various blockchain-based security applications based on the area considered are proposed in ^38^ such as:

**•    Data trading and sharing**

Applications in this area deal with data as a commodity that enables vehicles to purchase and sell data from the network. Generally, this can be seen as the underlying conception of all blockchain-based IoV structures due to the demand to share data among vehicles to coordinate, such as computational information for resource sharing. The data in vehicular networks can be classified into four categorizations include exchanged messages, user personal data, and user behavioral information among vehicles, besides vehicles’ trust rating values, which evaluate the user’s trustworthiness by other nodes on the reliance of their previous history.

**•    Transportation**

Applications in this area mainly concentrate on real-time vehicle, movement, coordination, and management to enable more efficient transportation. It launches beneficial collaboration opportunities for centralized and intensive computation such as ridesharing/carpooling, platooning, and smart parking applications.

**•    Smart grid**

The application in this area deals with Electric Vehicles (EVs) that need to be fault-tolerant and promptly handle any possible faults. The widespread known application for smart grids is securing EV charging and providing a platform to reward vehicles that share and supply the grid with extra energy.

**•    Resource sharing**

This application facilitates the trading of computational resources in exchange for money while taking the complexity of automotive networks and edge computing into account. Instead of relying on a single third party, it can create a distributed open market system. In such applications, vehicles can be both producers and consumers of computing resources, buying resources from the RSUs. It is assumed that, in contrast to traditional embedded systems, vehicles may be supplied with comparatively higher processing capabilities.

*Categorization based on security features*

The various blockchain-based security solutions are classified according to ^38^ into the security requirements met, protection against network-specific security attacks and authentication techniques, and security proofs, which are mentioned without any details. The following summarizes the first three categories:

1.  Security requirements

To alleviate attacks and vulnerabilities in a network, the network should meet a security requirement condition. Some of these security constraints are implicitly provided by blockchain technology due to its decentralized structure and tamper-proof storage mechanism. Therefore, all blockchain-based security applications will by default fulfil the following security requirements which include decentralization, immutability, unforgeability, and traceability. On the other hand, to increase its robustness, other security requirements for blockchain-based applications must be considered, excluding those provided by blockchain naturally including non-repudiation and scalability enhancements. The following explains both security requirements classifications based on the inherited blockchain features or the acquired ones based on ^38^.

**a.  Inherited features:**

- **Decentralization** which several applications in vehicular networks have utilized, such as decentralized communication, data sharing, and identity management, to enable a direct communication or P2P network independently and then transactions are verified by some of the dedicated blockchain nodes that helps in preserving the vehicles’ privacy.
- **Tamper-resistant** feature for all data recorded in the blockchain that confirms immutability since tampering data of one block will adjust its hash value and then will be removed from the chain.
- **Unforgeability** enables networks to prevent forging data or a user’s digital signature by malicious nodes to personate others. For example, using a blockchain-based standard authentication procedure called Zero-Knowledge Proof ensures unforgeability.
- **Traceability** through the cryptographic hash can assist the application's targets in tracking any adverse behavior or malicious broadcasting message to prevent uncertainty and collisions in vehicular networks.
- **Public audits** with the help of implementing consensus mechanisms that have been used for authentication purposes such as assessing transnational data trading in the vehicular network publicly.

**b.  Acquired features:**

On the other hand, other security requirements for blockchain-based applications are taken into account, excluding those provided by blockchain naturally, to improve its robustness. Some of these security requirements are ^38^:

- **Non-repudiation-based applications** make sure that senders cannot refuse the message's transmission, and that it is easy to identify the dedicated vehicle in the event of an accident. One attack taken into consideration for this security requirement is the mitigation of repudiation attacks.
- **Privacy preservation-based applications** assure that the drivers' and participating nodes' private information are protected from unauthorized observers beside maintain confidentiality from the public or adverse parties. For instance, conditional link-ability or traceability via conditional privacy would allow a government agency or other reliable party to reveal a vehicle's true identity if it is discovered to be disruptive and prevent "double claim" attacks.
- **Wallet security-based applications** support the majority of vehicular applications that use a reward system to encourage network cooperation between vehicles or that allow vehicles to trade cryptocurrencies for data and services. Therefore, each user of a vehicle has an associated e-wallet that protects the wallet from harmful attacks. It can be achieved by ensuring the safety of vehicles' accounts using an encrypted digital signature that forbids adversaries from accessing the wallets without the necessary keys and certifications.
- **Scalability enhancement-based applications** ensure that the network is adequate even when many vehicles are utilizing it. The scalability of a blockchain is determined by how well it can handle high transaction volumes in a blockchain-based environment. One option to satisfy this criterion is to use a dynamic consensus mechanism with low computational overhead that adjusts to the traffic flow.
- **Low latency and high throughput requirements-based applications** are debatable and important when it comes to network security since a slow network provides attackers with more time to launch attacks.

2.  Authentication techniques

Authentication is an essential part of any security solution. It is considered the first line of defense against various vehicular networks’ attacks, as discussed in the security requirements in vehicular networks in Section III-A. The popular authentication techniques used in blockchain-based vehicular security applications are categorized into ^38^:

a. **Mutual/two-way authentication** which, based on its name, enables the client (vehicle) and access point (RSU) to validate each other. The access point is certain that it is initiating a session with an authorized client and the client confirms that it is opening the session with a legitimate access point.

b. **Anonymous or privacy-preserving authentication** which utilizes the anonymity of the vehicles is protected during the authentication process. It can be divided into five groups, Public Key Infrastructure (PKI) and symmetric cryptography-based methods besides identity-based signatures, certificate-less signatures, and group signatures schemes.

c. **Certificate-based authentication** with an associated digital certificate, the first node can authenticate itself to the second node. A digital certificate's owner can be verified with the use of a digital signature on the certificate, and it also contains the public key for the associated entity. In the case of vehicular networks, the digital signature is confirmed by a trusted authority (TA).

d. **Batch verification** enables verifying a group of messages or nodes, instead of verifying just one message or item at a time. When confirming several messages, it can assist security techniques in overcoming the time delay. Three categories can be derived out of it: different signers signing the same message, different signers signing different messages, and verification that a signer signs a separate message.

e. **Zero-Knowledge Proof (ZKP)** is utilized when two nodes need to be authenticated without exchanging confidential information. In general, it meets the two fundamental security features of soundness and zero-knowledge, which guarantee that no information is leaked during the proof and that the verifier would never accept an improper result.

f. **Threshold authentication** is a common procedure for demonstrating the dependability of messages in a vehicular network. The receiver node can accept a message when a certain quantity of vehicles in the network confirms it, for instance by utilizing a threshold ring signature mechanism.

More details in ^38^ can be found, and a table called VI lists the references for various security frameworks and authentication techniques.

3.  Network-specific security attacks

Vehicular networks are vulnerable to several security attacks due to their open and dynamic operating environment. Hence most security schemes are proposed frameworks that resist well-known network security attacks. Attacks such as reply, spoofing, impersonation, and Sybil. The list of fourteen common network-specific security attacks, their definitions, the related security issues, and some references for security mechanisms proposed in vehicular networks are listed and summarized in a beneficial table in ^38^.

## Challenges and possible future research directions

### Integration challenges of blockchain within vehicular networks

The main six challenges that need to be addressed when incorporating blockchain into the IoV with edge computing scenarios and the significant challenges faced in implementing blockchain-based applications for securing vehicular networks are summarized below based on ^8, 38^, which conduct more discussion about these challenges, besides highlighting the potential solutions to address them in other literature can be found in.

*1.  Security and privacy*

By guaranteeing data immutability, blockchain technology can be integrated into vehicle networks to provide default security measures and prevent data manipulation. However, because blockchain is built on several methodologies, it cannot immediately ensure security and anonymity. To guarantee the security of the block contents as well as the nodes' privacy, methods including contemporary cryptographic techniques, pseudonyms, anonymous reputation systems, and off-chain storage are required. For instance, sensitive information like journey directions is offloaded for multiple tasks when the vehicular nodes are employed for edge computing. Therefore, in order to restrict access by unauthorized individuals, such information must be encrypted. However, cypher-text slows down the analytics process, necessitating a speed-up solution in the blockchain architecture for edge computing that protects privacy. Additionally, the sensitive nature of the given data is necessary to protect user privacy and preserve transparency. To balance privacy and transparency in a distributed ledger, encryption and complementary access control methods are required.

*2.  Performance “latency, energy consumption, throughput, and scalability.”*

The direct integration of blockchain technology into vehicular networks may be constrained by requirements such as the ability to handle a massive amount of data and transactions in an environment of extreme mobility. Therefore, in addition to the significance of security and privacy, the blockchain-based vehicular network's performance metrics are also crucial. These matrices include scalability, throughput, usage of energy, and latency. The throughput, or number of transactions validated per second, is a numerical indicator of the blockchain system's scalability. Generally, greater scales can boost lower throughputs by appropriately altering the algorithm. The blockchain must scale up to a very high level due to the latency-critical environment in vehicular networks. Different modern standards and protocols created for Bitcoin or cryptocurrency applications are less time-sensitive than the desires for vehicular networks. Therefore, sharding and off-chain scaling are complementary methods of increasing a blockchain's throughput which is debatably required. These technologies, however, are still in the early stages and require help from other methodologies, for instance, when off-chain methods are required. Therefore, the goal of future research should be improving transaction throughput while lowering bandwidth, storage, and computing power needs without compromising security.

*3.  Vehicular specified and optimized consensus*

The standard and productive consensus algorithms are mainly elaborated for cryptocurrency applications. Therefore, the discrete nature of vehicular networks creates new challenges regarding applying these consensuses in such kinds of networks including security, validating a block, and consuming energy, besides utilizing incentive mechanisms and penalty methods.

Choosing the appropriate consensus algorithms and blockchain type can enhance the performance. For instance, DPoS significantly improves transaction throughput compared to PoS. Furthermore, consortium and private blockchains are more competent regarding the blockchain type than permissionless blockchains because there are fewer validator nodes. Since consortium and private blockchains are thought to be more centralized than permission-less blockchains, it is important to carefully consider the trade-off between decentralization and throughput. To expand the applicability of integrating the blockchain into the vehicular environment, it is also necessary to consider the optimized and vehicle-specific consensus processes.

*4.  Incentive mechanisms*

In vehicular networks, applications built on public and consortium blockchains often rely on participation from one or more nodes. This contribution contains newly created block validation, the creation of relevant information, and blockchain recording. Rewarding contributors will therefore be essential to accelerating blockchain-related schemes. For vehicular applications, some incentive mechanisms are suggested, such as an incentive system based on pricing theory. In addition, a special consensus method is presented to support vehicular applications such as a Proof of- Reputation (PoR), where the validator nodes are chosen based on their reputations to improve the blockchain network security.

*5.  Quantum computing attacks*

In the coming years, Quantum computing is a research field that is expected to have broad applications. However, cryptography in blockchain depends on one-way hashing techniques, which may not be as secure as they are present with the emergence of quantum computing. Furthermore, quantum computers offer an incredibly unique computational power scale. Thus, only a few quantum computers may easily defeat the conventional blockchain network's computing power. Consequently, vehicular applications must be quantum-resistant, or they will be vulnerable to 51% and byzantine attacks. Attempts to attack the vehicular network using quantum computers destroy trust in the blockchain network and aim to damage some components of the network rather than just a single node. Research on quantum attacks is ongoing, and several solutions are being proposed.

*6.  Attacks on blockchains*

Blockchain technology's unique nature creates several new attacks not considered in conventional centralized systems. These attacks include 51% attacks, selfish mining, eclipse attacks, DNS attacks, crypto-jacking, and more. Even though blockchain offers useful features like decentralization and transparency for vehicular edge computing frameworks, it is still vulnerable to several attacks. More importantly, the network can be forged, and the transactions may be manipulated when a significant percentage of participating nodes are compromised. Thus, to incorporate various blockchains in vehicular networks, a scalable and reliable consensus mechanism is needed. 

In conclusion, despite the enormous potential of using blockchain and the integration of edge computing systems, it faces many difficulties that may restrict its widespread adoption, primarily decentralization, scalability, and security. Scalability impacts the capacity to process transactions, whereas decentralization permits the network to be permission-less without centralized supervision. Currently, the practical viability of any blockchain-based solutions is impeded by scalability problems, low throughput restrictions, excessive latency, and overburdened resources. Additionally, even though security encourages immutability and resistance to attacks, there are new security concerns brought on by outsourcing or offloading services at the edge that require further research. Additionally, self-organization decreases management complexity by introducing autonomous procedures, but it also creates more security concerns ^19^. Additionally, the blockchain and edge computing network integration, storage, and computation functionalities should be deeply investigated with flexibility and stability from multiple points of view. Consequently, different aspects related to resource management need comprehensive research efforts to be broadly tackled. Finally, new methodologies like the directed acyclic graph, big data, and artificial intelligence will promote the combination of blockchain and edge computing ^19^.

### Possible future research direction

The following are four main challenges facing the efficiency of blockchain technology and vehicular network integration, which consequently will affect vehicular computation offloading. 

*1.  Privacy and security*

For any network, including computation offloading in vehicular networks, security and privacy are major concerns. Numerous works have highlighted multiple security risks and provided recent solutions. Devices at the network's edges are often subject to the same security and privacy standards that apply to cloud computing. The edge, however, deals with a lot more sensitive data. Vehicles, for instance, broadcast information about their regular trips and private information may be sent to unreliable servers. Thus, issues such as remote computation, context-aware computing, and remote data access make privacy and security challenges in VEC. 

The privacy of the data that has been offloaded can be adequately increased with an effective trust management system and data isolation mechanism. A more effective anonymous authentication method can also be integrated with VEC. Examining possible underlying threats and vulnerabilities during the offloading process is required to maintain secure communication and guarantee the confidentiality and privacy of data. Moreover, to satisfy the safer driving object, providing reliable data is necessary to prevent exchanging false information. However, it is difficult to create integrated security and privacy rules since there is no centralized authority. Consequently, protections for privacy and security are therefore essential requirements that must be met at the network edge. There is a demand for additional investigations, research, and solutions in this field due to this importance ^16, 20^.

*2.  Advancement of vehicular communication and its supportive technologies*

*VEC/5G integration*

5G networks will provide significant support for intensive applications. However, such intensive applications need high computation resources, which constrained 5G devices’ resources inappropriately. As a result, VEC will be able to facilitate computation-intensive 5G applications ^41^.

*Advancement of UAV edge node*

UAVs have a major impact on autonomous systems and the automotive sector. By addressing the issue of computing performance through data offloading, UAV integration with MEC or edge computing has successfully increased the efficiency of constrained OBU resources ^20^.

*3.  Efficient algorithms development*

Regarding the optimization algorithms in partitioning, scheduling, and data retrieval for computation offloading, solutions constructed on machine learning emerge as a viable alternative for offloading processes in VEC systems. Furthermore, making decisions in such approaches will be logically based on the current network situations rather than the standard mathematical model, based on measures such as energy, delay, throughput, and cost ^41^.

*4.  Enhancement scalability*

Basically, vehicular networks are large-scale in the real world. Thus, providing network scalability is required due to the massive increase in vehicles and their requested services. Furthermore, maintaining a consistent and acceptable performance level regarding users’ QoS/QoE is needed to provide network scalability for supporting an enormous traffic load. Therefore, clustering and load balancing are two terms to be exploited to fulfil vehicular applications effectively ^39^.

### Blockchain edge of vehicle (BEoV)

One of the best ways to meet the security needs of the Edge of Things (EoT) through transparent transactions is with blockchain technology. By using blockchain, vulnerabilities like identity theft, DDoS attacks, tampering with transactions, and user privacy breaches can be prevented ^21^. With less latency and real-time analytics/recommendations, the usage of blockchain in the IoT has the potential to bring forth a new era where developers of mobile applications may give users secure, transparent, irreversible, and decentralized applications ^21^. Therefore, the most recent beneficial survey ^21^ proposes a new paradigm called blockchain for the edge of thing or BEoT, and presents various applications in five domains in-depth such as smart transportation domains. Moreover, it analyzes security challenges and explores four essential security services enabled by blockchain, including access authentication, data privacy, attack detection and trust management.

**Figure 6.**
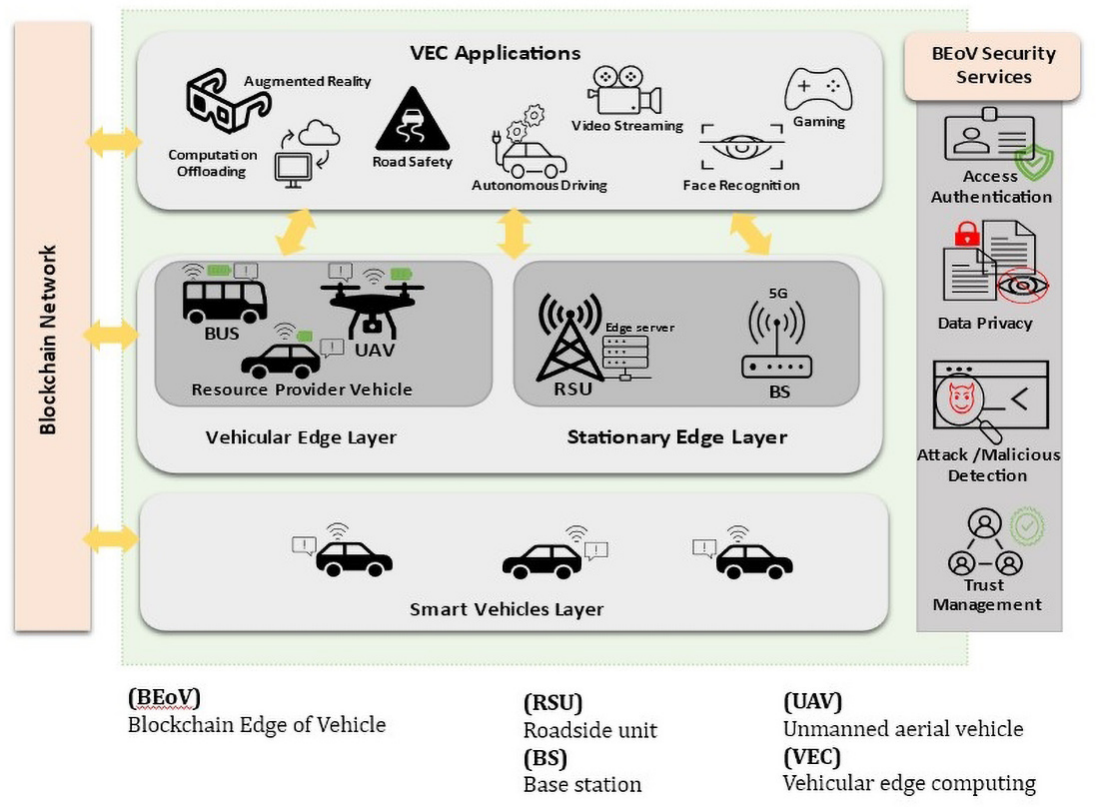
Blockchain Edge of Vehicle (BEoV) structure and the provided blockchain security services.

Established on these blockchain features, the promising paradigm (BEoT), and the ability to integrate blockchain technology into the VEC, we can introduce a new concept called blockchain edge of vehicle (BEoV). We can use the BEoV paradigm for describing the incorporation of vehicular edge computing VEC with blockchain technology that enables blockchain-based security services for the Intelligent Transportation System ITS specifically. Application scenarios such as computation/task offloading, augmented reality, autonomous driving, face recognition and most intensive vehicular applications can leverage integrating blockchain to improve the security of all these applications that need highly intensive computing resources. The authors in ^21^ state that blockchain can offer essential security services for BEoV, including access authentication, data privacy, attack detection, and trust management. According to what the authors propose in ^21^, and based on our suggestion for VEC applications particularly, the following figure illustrates the structure of integrating the blockchain into VEC to provide blockchain security services ( Figure 6).

Various applications study the integration of blockchain technology by considering all benefits of the above-mentioned attractive features ^8^. For instance, blockchain advances the computation offloading security in edge computing compared to offering security solutions such as access control. In addition, incorporating blockchain does not solely manage the computation offloading efficiently but also allows trustworthy authentication on different offloading tasks to provide decentralization protection for resources ^62^. However, although some research has been carried out on securing computation offloading in the vehicular network, only a few studies have investigated adopting blockchain technology ^20^.

Particularly for vehicular network applications, two valuable features can be leveraged. First, the blockchain technology itself and its structure are designed to offer security features and data reliability independently with no need for a trusted authority. Second, the functionality of smart contracts provides the ability to perform complex programmable assignments and allows intelligent communication among a massive number of entities ^38^. Consequently, many models are developed to ensure security, protect privacy, and establish trust management in vehicle networks. However, blockchain technology is currently being considered a crucial sector of several vehicular network approaches that provide a safe, private, and reliable environment ^37^. 

## Conclusions

This review paper provides a comprehensive overview and discussion regarding the promising move to ITS efficiently and securely. It provides a comprehensive review of recent related surveys and some current related studies. Then it gives a background of some preliminary topics related to VEC and computation offloading besides an overview of most related topics to blockchain technology including consensus mechanisms and smart contracts. After that, a comprehensive discussion regarding security, privacy and trust problems in the vehicular network and computation offloading applications is discussed. Additionally, explaining the motivation of integrated blockchain technology as a proposed distributed security solution and introducing the BEoV framework that provides security services based on blockchain. Regardless of all current research efforts, significant research challenges and open issues are still facing the computation offloading in the context of vehicular environments, especially data security and privacy preservation and trust management issues of computation offloading in blockchain-based VEC are still not fully discussed in most of the existing works since most of the existing research focus on the task offloading algorithm. Thus, it proposes a new solution based on trust management given by our new paradigm BEoV, which offers various security services and trust management one of them, as our future work. As a result, we are working on enhancing the data security communication and raising the offloading computation successful rate in case of the need to offload tasks in the VEC environment into other vehicles by leveraging blockchain security features *i.e.*, BEoV and 5G cellular network technology for high-speed connectivity. The goal is to integrate blockchain technology to manage the trust level of all nodes participating in the computation offloading processes based on a reputation calculation mechanism in addition to maintaining the latency requirements, energy consumption, and scalability issues besides meeting the security goals. The proposed mechanism will be considered mainly as a strengthening factor of the security and reliability of computation offloading tasks, encouraging techniques for honest vehicles to contribute, and mitigating the method of malicious ones from contribution by leveraging exclusively one of the BEoV security services which is trust management. It also aims to maintain the quality of performance and improves the success rate of completion of the computation offloading tasks. Moreover, most surveys recommended employing emerging technologies like edge computing, 5G, SDN, AI, and ML algorithms, such as reinforcement and deep learning, since blockchain can be used in a hybrid vehicular, thus, as a second phase, UAVs or drones, ML, verifiable computing, and new cryptography technique will be considered in contemplation of making the system more robust.

## Ethics and consent

Ethical approval and consent were not required.

## Data Availability

No data are associated with this study.
