# Supplementary material for: A survey of security, privacy and trust issues in vehicular computation offloading and their solutions using blockchain
Source: Open Res Eur. 2023 Oct 25;3:110. Originally published 2023 Jul 7. [Version 2] doi: 10.12688/openreseurope.16189.2 (PMC10646339; doi:10.12688/openreseurope.16189.2)
Supplement: Peer review history

# Reviewer report: Reviewer response for version 2

**DOI:** 10.21956/openreseurope.18070.r35834

**Published:** 2023-11-14

**Authors:** Omprakash Kaiwartya

**Affiliations:** 1 Department of Computer Science, Nottingham Trent University, Nottingham, England, UK

My comments on the 1st round review have been effectively incorporated in the revised paper. I have no further comments, and I am happy to make a recommendation for indexing.

Is the review written in accessible language?

Yes

Are all factual statements correct and adequately supported by citations?

Yes

Are the conclusions drawn appropriate in the context of the current research literature?

Yes

Is the topic of the review discussed comprehensively in the context of the current literature?

Yes

Reviewer Expertise:

Vehicular networking/Connected vehicles

I confirm that I have read this submission and believe that I have an appropriate level of expertise to confirm that it is of an acceptable scientific standard.

# Reviewer report: Reviewer response for version 1

**DOI:** 10.21956/openreseurope.17476.r34927

**Published:** 2023-09-18

**Authors:** Marco Gramaglia

**Affiliations:** 1 Universidad Carlos III de Madrid, Getafe, Community of Madrid, Spain

This paper provides a comprehensive survey of many aspects related to Vehicular Edge Computing. The authors do meticulous work in reviewing the current efforts available in the literature (including mentioning other surveys).

 The paper addresses privacy and security aspects; an important topic such as privacy in machine learning with vehicular sensor data.

Is the review written in accessible language?

Yes

Are all factual statements correct and adequately supported by citations?

Yes

Are the conclusions drawn appropriate in the context of the current research literature?

Yes

Is the topic of the review discussed comprehensively in the context of the current literature?

Yes

Reviewer Expertise:

machine learning applied to networks

I confirm that I have read this submission and believe that I have an appropriate level of expertise to confirm that it is of an acceptable scientific standard.

# Response

**Published:** 2023-09-26

**Authors:** Sharifah Fayi

**Affiliations:** School of Engineering and Informatics, University of Sussex, Brighton, England, UK

Thanks for your time to review my article. I appriciate it!   Kind regards  Sharifah

# Reviewer report: Reviewer response for version 1

**DOI:** 10.21956/openreseurope.17476.r33625

**Published:** 2023-09-18

**Authors:** Omprakash Kaiwartya

**Affiliations:** 1 Department of Computer Science, Nottingham Trent University, Nottingham, England, UK

The paper presents a critical review of vehicular edge computing and related security risks and solutions. I find the article comprehensive, and well-written. However, the following points can further improve the article:

1. In the introduction, the paper should be summarized in terms of sections considered and their significance. 
2. The taxonomy of literature is missing from this, which is very urgent for the readability of this paper. 
3. The paper is lacking in terms of figure-based explanation. The number of figures used is less considering its a review article. More figures should be added. 
4. Table based summary for comparisons of literature is also missing in the paper. It should be added in several subsections in the paper. 

Is the review written in accessible language?

Yes

Are all factual statements correct and adequately supported by citations?

Yes

Are the conclusions drawn appropriate in the context of the current research literature?

Yes

Is the topic of the review discussed comprehensively in the context of the current literature?

Yes

Reviewer Expertise:

Vehicular networking/Connected vehicles

I confirm that I have read this submission and believe that I have an appropriate level of expertise to confirm that it is of an acceptable scientific standard, however I have significant reservations, as outlined above.

# Response

**Published:** 2023-09-26

**Authors:** Sharifah Fayi

**Affiliations:** School of Engineering and Informatics, University of Sussex, Brighton, England, UK

Thanks for your time to review my article. I appriciate it! I will work on revising the aricle and make changes accordingly to the provided comments.  Kind regards  Sharifah
